# Structural basis for activation of the growth hormone-releasing hormone receptor

**DOI:** 10.1038/s41467-020-18945-0

**Published:** 2020-10-15

**Authors:** Fulai Zhou, Huibing Zhang, Zhaotong Cong, Li-Hua Zhao, Qingtong Zhou, Chunyou Mao, Xi Cheng, Dan-Dan Shen, Xiaoqing Cai, Cheng Ma, Yuzhe Wang, Antao Dai, Yan Zhou, Wen Sun, Fenghui Zhao, Suwen Zhao, Hualiang Jiang, Yi Jiang, Dehua Yang, H. Eric Xu, Yan Zhang, Ming-Wei Wang

**Affiliations:** 1 The CAS Key Laboratory of Receptor Research, Shanghai Institute of Materia Medica, Chinese Academy of Sciences, 201203 Shanghai, China; 2 The National Center for Drug Screening, Shanghai Institute of Materia Medica, Chinese Academy of Sciences, 201203 Shanghai, China; 3 Department of Biophysics, and Department of Pathology of Sir Run Run Shaw Hospital, Zhejiang University School of Medicine, 310058 Hangzhou, China; 4 School of Pharmacy, Fudan University, 201203 Shanghai, China; 5 iHuman Institute, ShanghaiTech University, 201210 Shanghai, China; 6 State Key Laboratory of Drug Research, Shanghai Institute of Materia Medica, Chinese Academy of Sciences, 201203 Shanghai, China; 7 Drug Discovery and Design Center, Shanghai Institute of Materia Medica, Chinese Academy of Sciences, 201203 Shanghai, China; 8 University of Chinese Academy of Sciences, 100049 Beijing, China; 9 School of Life Science and Technology, ShanghaiTech University, 201210 Shanghai, China; 10 School of Basic Medical Sciences, Fudan University, 200032 Shanghai, China

**Keywords:** G protein-coupled receptors, Cryoelectron microscopy, Cryoelectron microscopy

## Abstract

Growth hormone-releasing hormone (GHRH) regulates the secretion of growth hormone that virtually controls metabolism and growth of every tissue through its binding to the cognate receptor (GHRHR). Malfunction in GHRHR signaling is associated with abnormal growth, making GHRHR an attractive therapeutic target against dwarfism (e.g., isolated growth hormone deficiency, IGHD), gigantism, lipodystrophy and certain cancers. Here, we report the cryo-electron microscopy (cryo-EM) structure of the human GHRHR bound to its endogenous ligand and the stimulatory G protein at 2.6 Å. This high-resolution structure reveals a characteristic hormone recognition pattern of GHRH by GHRHR, where the α-helical GHRH forms an extensive and continuous network of interactions involving all the extracellular loops (ECLs), all the transmembrane (TM) helices except TM4, and the extracellular domain (ECD) of GHRHR, especially the N-terminus of GHRH that engages a broad set of specific interactions with the receptor. Mutagenesis and molecular dynamics (MD) simulations uncover detailed mechanisms by which IGHD-causing mutations lead to the impairment of GHRHR function. Our findings provide insights into the molecular basis of peptide recognition and receptor activation, thereby facilitating the development of structure-based drug discovery and precision medicine.

## Abstract (web summary)

Growth hormone-releasing hormone (GHRH) controls metabolism and tissue growth through binding to the cognate receptor (GHRHR). Here authors report the structure of the human GHRHR bound to its endogenous ligand and the stimulatory G protein which reveals a characteristic hormone recognition pattern of GHRH by GHRHR.

## Introduction

Class B G-protein-coupled receptors (GPCRs) are key players in hormonal homeostasis and important drug targets for endocrinal and neuronal disorders. Growth hormone-releasing hormone receptor (GHRHR), a prototypical class B GPCR, is expressed by somatotropic cells of the pituitary gland. Activation of GHRHR by GHRH, a 44-amino acid peptide released by the hypothalamus^1,2^, results in the secretion and production of growth hormone (GH) through cyclic adenosine monophosphate (cAMP)-dependent pathways^3^. Numerous studies demonstrated that GHRH exerts a variety of bioactivities due to its wide distribution and autocrine/paracrine mechanisms^4,5^. Therefore, GHRH and its analogs, including tesamorelin, MR-409, JI-38, and MIA-690, have been developed as potential therapeutic agents to treat diabetes, cancers, and cardiovascular diseases^5–9^.

Like other class B GPCRs, GHRHR consists of an extracellular domain (ECD) and a seven-transmembrane helix domain (7-TMD)^10,11^. Recently published cryo-electron microscopy (cryo-EM) structures of class B GPCRs bound to a G_s_ heterotrimer protein include parathyroid hormone receptor 1 (PTH1R), glucagon-like peptide-1 receptor (GLP-1R), calcitonin receptor, calcitonin gene-related peptide receptor (CGRPR), two subtypes of corticotrophin-releasing factor (CRF1R and CRF2R), and adrenomedullin receptors (AM1R and AM2R), as well as pituitary adenylate cyclase-activating polypeptide (PACAP) type I receptor (PAC1R) and vasoactive intestinal polypeptide receptor (VIP1R), revealing a common mode of ligand-induced receptor activation^10–19^. The C-terminal α helix of peptide ligand recognizes and binds to the ECD, thereby allowing its N-terminus to interact with the extracellular TM core. This is followed by a major conformational change that involves a large kink at the TM6 to open the intracellular face for G protein coupling^15^. However, ligand-binding specificity and roles of ECD in receptor activation vary widely among class B GPCRs due to diverse amino acid sequences of both peptidic ligands and receptors^15,20^.

Here, we employed the single-particle cryo-EM approach to determine the near-atomic resolution structure of the human GHRHR bound to GHRH in complex with a heterotrimeric G_s_ protein. Together with functional studies and molecular dynamics (MD) simulations, our results provide key insights into the structural basis of ligand recognition, receptor activation, and isolated growth hormone deficiency (IGHD) causing mechanism related to GHRHR, thereby offering a template for rational design of drugs against this receptor.

## Results

### Structure determination of the GHRH–GHRHR–G_s_ complex

**Fig. 1:**
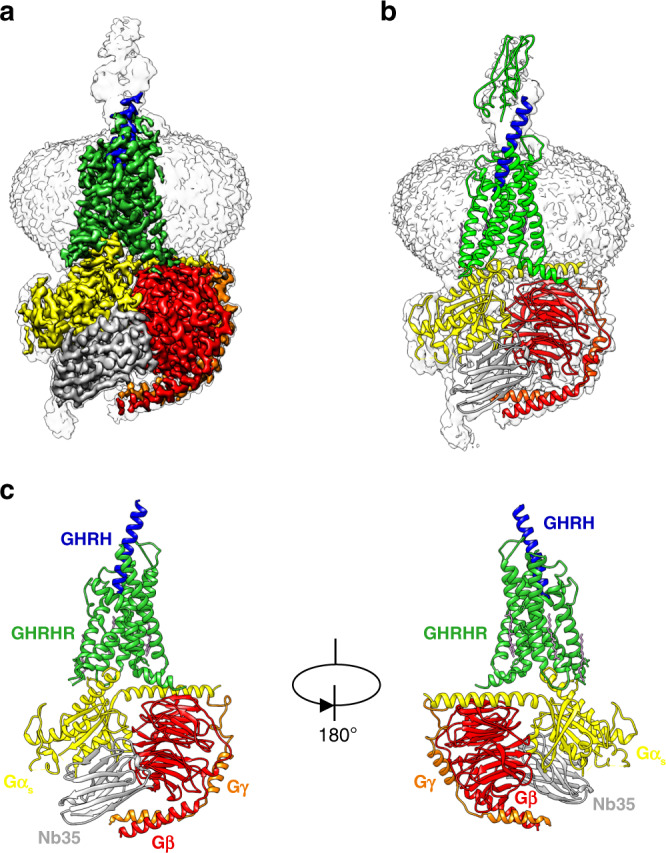
The overall cryo-EM structure of GHRH–GHRHR–G_s_ complex. **a** Cryo-EM density map that illustrates the GHRH–GHRHR–G_s_ complex and the disc-shaped micelle. The unsharpened cryo-EM density map at the 0.005 threshold shown as light gray surface indicates a micelle diameter of 10 nm. The colored cryo-EM density map is shown at 0.028 threshold. **b** GHRH–GHRHR–G_s_ complex model and GHRHR ECD crystal structure model docked into the cryo-EM map. **c** Cartoon representation of the GHRH–GHRHR–G_s_ complex is shown with annular lipids in purple stick representation. Lime green, GHRHR; blue, GHRH; yellow, G_s_ Ras-like domain; red, Gβ; orange, Gγ; gray, Nb35; plum, lipid, and cholesterol.

We developed a NanoBiT tethering strategy to stabilize the assembly of a GHRH–GHRHR–G_s_ complex for cryo-EM studies, overcoming the lack of stability of the above complex (Supplementary Fig. 1 and Supplementary Table 1), as it has been used for the VIP1R-G_s_ complex^19^. Using this approach, we were able to obtain a GHRH-GHRHR–G_s_ complex with improved homogeneity and stability (Supplementary Fig. 2). The GHRH–GHRHR–G_s_ complex was vitrified and cryo-EM images were collected under a Titan Krios microscope equipped with K2 summit direct detector. The structure of GHRH–GHRHR–G_s_ complex was determined from 307,018 particles to an overall resolution of 2.6 Å (Supplementary Figs. 3 and 4). The resulting model contains 28 residues of GHRH (residues 1–28), Gαβγ subunits except the α-helical domain (AHD) of Gα_s_, and GHRHR residues from 119 to 394. Besides, the ECD region of GHRHR was not resolvable with this limited dataset, perhaps reflecting its highly dynamic and conformationally flexible property when bound to GHRH. We rigid-body fitted the GHRHR ECD (residues 25–118) crystal structure (PDB accession: 2XDG) to the low-pass filtered map (Fig. 1b). The majority of amino acid side chains were well resolved in the final model (Supplementary Fig. 5), which was refined against the EM density map (Fig. 1a) with excellent geometry (Supplementary Table 2). Owing to the high-resolution map, we identified one water molecule in the orthosteric binding site, and two water molecules in the G protein engaging pocket. Akin to cryo-EM structure of PTH1R–G_s_ complex, the TMD of GHRHR is surrounded by annular detergent micelle with a diameter of 10 nm, mimicking the natural phospholipid bilayer. Within the micelle, one bound cholesterol and two lipids are also clearly visible in the cryo-EM map.

### Molecular recognition of GHRH by GHRHR

**Fig. 2:**
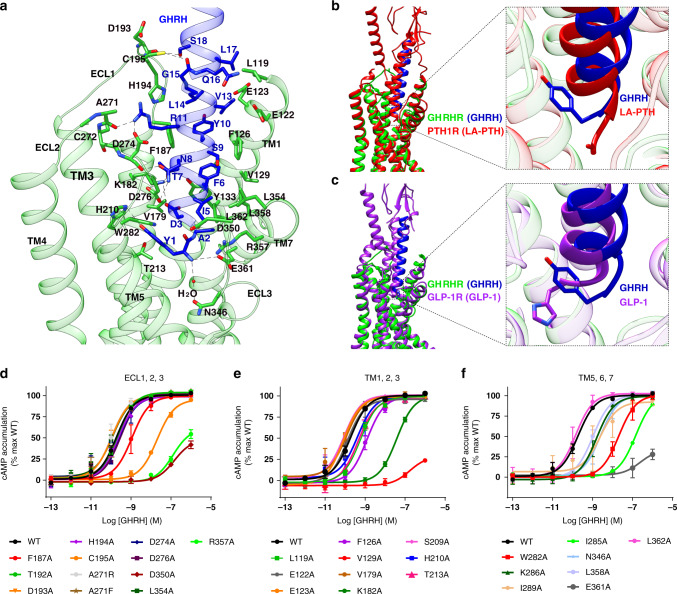
Molecular recognition of GHRH by GHRHR and comparison of that with LA-PTH–PTH1R and GLP-1–GLP-1R. **a** Detailed interaction between GHRH and the TMD pocket of GHRHR with hydrogen bonds shown as dotted lines. **b**, **c** Pairwise comparison of GHRH bound to GHRHR with GLP-1 and LA-PTH in complex with their corresponding receptors, showing the relative positions of peptide ligands. Lime green, GHRHR; blue, GHRH; red, PTH1R and LA-PTH; purple, GLP-1R and GLP-1. **d**–**f** The effects of mutation in the ligand-binding pocket on cAMP accumulation. cAMP levels were measured in wild-type (WT) receptor and alanine mutants in ECL1, 2, and 3 (**d**), TM1, 2, and 3 (**e**), and TM5, 6, and 7 (**f**). cAMP signals were normalized to the maximum response of WT and concentration–response curves were analyzed using a three-parameter logistic equation. Data shown are means ± S.E.M. of at least three independent experiments (*n* = 3–5), conducted in triplicate. Source data are provided as a Source data file.

In the complex structure, GHRH adopts an α-helical configuration when engaged with GHRHR. Compared to GLP-1 (ref. ^10^) and long-acting parathyroid hormone analog (LA-PTH)^15^, GHRH binds to the GHRHR TMD through a more extensive and continuous network of interactions involving all the extracellular loops (ECLs), all the TM helices except TM4, and the linker connecting ECD and TMD. The N-terminus of GHRH inserts deeply into the TMD core and engages an extensive set of receptor-specific interactions (Fig. 2a–c and Supplementary Table 3). Interestingly, Tyr^1P^ locates in the equivalent position of the second residue in other class B GPCR peptidic agonists, such as exendin-P5 (ExP5) and LA-PTH, where their first residues of the side chain have different orientations (Fig. 2b). The hydroxyl group of Tyr^1P^ forms hydrogen bonds with H210^3.37b^ (class B GPCR numbering in superscript)^21^ and van der Waals interactions with T213^3.40b^ and W282^5.36b^, whereas the main chain NH of Tyr^1P^ forms hydrogen bond with R357^7.38b^ of TM7 and contacts a water molecule that connects with N346^6.57b^ of TM6-ECL3 hinge, possibly stabilizing the ECL3 in an active state. This observation is consistent with our observation that impairing these contacts dramatically decreased the potency of GHRH in stimulating cAMP accumulation (Fig. 2d–f and Supplementary Table 4). The most conserved Asp/Glu at position 3 across peptide hormones of the glucagon receptor (GCGR) subfamily, Asp^3P^ in the case of GHRH, forms salt bridges with K182^2.67b^, which is further strengthened by a polar network composed of Thr^7P^, Y133^1.43b^, and D183^2.68b^. From evolutional biology perspective, both Asp^3P^ and K182^2.67b^ are fully conserved for GHRH and GHRHR from dozens of species (Supplementary Fig. 6a–b). Indeed, D^3P^A and K182^2.67b^A diminished the potency of GHRH by ~4- and 200-fold (Fig. 2e and Supplementary Fig. 6c), respectively. The combined structural, pharmacological, and evolutional investigations point to a crucial role of Asp^3P^ in peptide binding and receptor activation. Phe^6P^ is another conserved residue and contributes extensive contacts with surrounding residues of GHRHR, including Pi–Pi stacking with F126^1.36b^ and Y133^1.43b^, and hydrophobic contacts with V129^1.39b^ and L362^7.43b^, where these four residues are either fully conserved or physiochemically similar among GHRHRs of different species. By forming salt bridges with D274^5.52b^ and hydrogen bonds with backbone atoms of H194^ECL1^ and A271^ECL2^, fully conserved Arg^11P^ greatly stabilizes the ECL1–ECL2 interface relative to the conserved TM3–ECL2 disulfide bond (Cys^3.29b^‒Cys^ECL2^) of class B GPCRs. This interface is further stabilized by close contacts between ECL1 and GHRH (T-shape Pi stacking between Tyr^10P^ and F187^ECL1^, and hydrogen bonds between Ser^18P^ and D193^ECL1^), as well as between ECL2 and GHRH (hydrogen bonds between Asn^8P^ and D274). For residues like Ala^2P^ and Ile^5P^, they contribute hydrophobic interactions with L362^7.43b^ and D350^ECL3^, which are conserved among different species. The remaining residues, including Ala^4P^, Ser^9P^, and Gly^15P^, are variable and receptive to substitution by Ala, Gly, Cys, or Ser, indicating their role of structural complementarily. Amino acids of the peptide after 20 may have rich interactions with the ECD as indicated by the model, in which the ECD was docked in the EM map as a rigid body (Fig. 1b). Indeed, cAMP signaling was nearly abolished in HEK 293 T cells expressing a truncated ECD construct, i.e., GHRHR(119–423), suggesting an essential role of ECD in GHRH recognition and receptor activation (Supplementary Fig. 7a). In our 1 μs MD simulation, the ECD of GHRHR was able to twist around the GHRH helix, while the TMD core is quite stable in a single dominant conformational state as seen previously with PTH1R^15^. Specifically, massive hydrophobic contacts between GHRH (R20, L22, L23, and M27) and GHRHR ECD (L34, L62, F82, Y108, P109, and L118), as well as several hydrogen bonds (Q24 of GHRH and C112 of GHRHR), were observed, which may stabilize the binding of GHRH (Supplementary Fig. 7b). These results are consistent with the findings on the same receptor reported in the literature^22,23^. In fact, such a dynamic and flexible feature of ECD could also be found among other class B GPCRs, such as PTH1R^15^, GLP-1R^10^, and VIP1R^19^, where the conversion from a closed conformation in the apo-state to an extended open conformation is required for activation (Supplementary Fig. 7c).

Unlike the helical extensions found in ECL1 of GCGR, GLP-1R, CRF1R, CRF2R, AM1R, and AM2R, and the unstructured ECL1 of PTH1R in active state, the ECL1 of GHRHR stretches around GHRH to form broad interactions. Consistently, disruption of GHRH-ECL interaction by F187^ECL1^A and C195^ECL1^A reduced GHRH potency by ~5- and 100-fold, respectively (Fig. 2d). Coupled with the conformational change from β-hairpin to α-helix in ECL1 of GCGR upon activation^24^, and the limited impact on G_s_-mediated cAMP signaling by alanine mutation on ECL1 of GLP-1R^25^, these results demonstrate the dynamic nature and diversified roles of ECL1 in class B GPCRs.

### Active structure of GHRHR

The overall arrangement of the GHRH–GHRHR–G_s_ complex is highly similar to GLP-1–GLP-1R–G_s_, glucagon–GCGR–G_s_, and LA-PTH–PTH1R–G_s_ complexes^10,14,15^. Superimposition of TMs shows that active GHRHR, GLP-1R, and PTH1R share similar folds with respect to the global conformation of the 7-TM bundle, as well as a similar organization of the extracellular end of TM6 and TM7 to accommodate peptide ligand. Except ICL3, other loop regions of the receptor are visible due to the high-quality cryo-EM density map, although these loops are relatively dynamic compared to the TMD bundle (Fig. 1c and Supplementary Fig. 5).

**Fig. 3:**
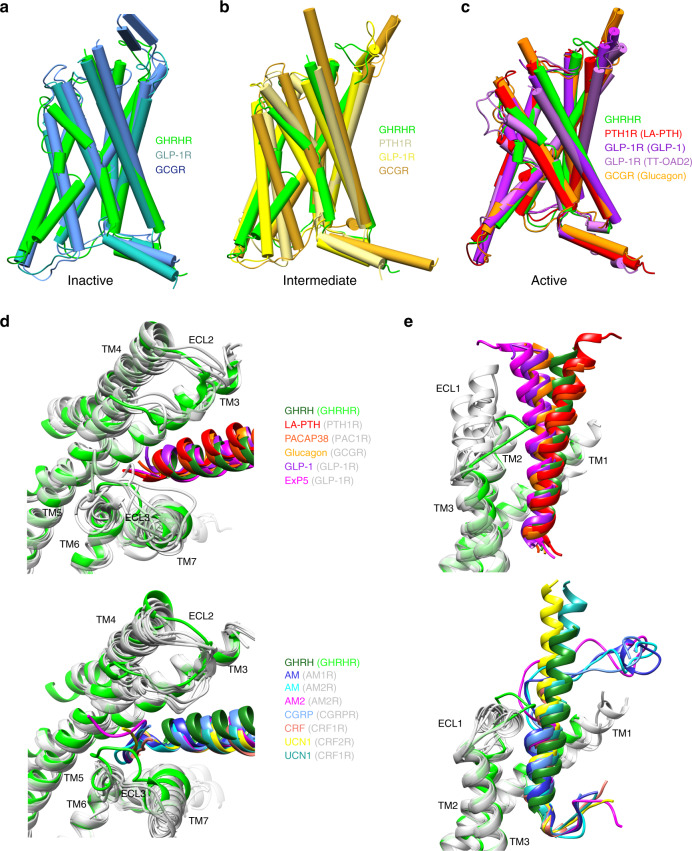
Structural comparison of active GHRHR with other class B GRCRs in active, intermediate, and inactive states. **a**–**c** Comparison of active GHRHR with inactive (**a**), agonist-bound intermediate (**b**), and both agonist- and G-protein-bound active (**c**) class B GPCRs. **d**, **e** Comparison of ligand conformations in G-protein-bound class B GPCRs; close-up view of the N-terminus (**d**), middle region (**e**), and C-terminus (**e**) of bound peptides in class B GPCRs.

Comparison of the GHRHR complex with inactive class B GPCR structures^26,27^, such as GLP-1R and GCGR, suggests that the most obvious conformational transformation is located at TM6 (Fig. 3a–c), where there is a large outward movement at the cytoplasmic face in the activated GHRHR structure upon coupling to G_s_. The TM6 outward displacement is correlated with the kink at the conserved Pro^6.47b^-X-X-Gly^6.50b^ motif^10,13,16,28^.

### Diversified peptide binding modes

Different from the ligand-binding pockets for small molecules in class A GPCRs, the peptide-binding pockets in class B GPCRs are larger and more extended, involving ECD, ECLs, and TMs. As shown in Fig. 3d, e, class B GPCR agonists adopt diversified conformations and orientations especially in the N- and C-terminals. Contrary to the single continuous helix observed in GHRH, GLP-1, glucagon, ExP5, PACAP38, and LA-PTH, the N-termini of AM, AM2, CGRP, CRF, and UCN1 looping back between TM5 and TM6, while AM, AM2, and CGRP also have a large kink at the C-terminal portion. Comparison of peptide recognition modes in the GCGR subfamily reveals that binding specificity mainly resides in three segments: N-terminus (first three residues in GHRH), middle region (6th to 11th residues), and C-terminal portion (Fig. 3d, e and Supplementary Fig. 8).

The orientation of the residues at N-terminus are different: the side chain of Tyr^1P^ in GHRH and His^1P^ in glucagon directly face TM3 forming hydrogen bonds with H210^3.37b^ and I235^3.40b^; the side chain of His^7P^ in GLP-1 directly faces TM5 and has cation–Pi interaction from upward R299^ECL2^; and the first two residues of LA-PTH (Ala^1P^Val^2P^) and ExP5 (Glu^1P^Leu^2P^) insert into the cleft between TM5 and TM6. Meanwhile, the orientation of highly conserved Glu/Asp^3P^ is significantly different (Supplementary Fig. 8d, e). These observations suggest the complexity of signal initiation.

The middle region of peptides has rich contacts with ECL1 and structurally flexible ECL2 in a peptide-dependent manner (Fig. 3e and Supplementary Fig. 8f). Resulted from longer α-helical extensions of TM2, the elevated ECL1 of GLP-1R and GCGR contribute additional contacts with the peptide C-terminal region. For receptors whose residues at 45.52 (adopted from GPCRdb numbering^29^, the second residue after the family-wide conserved cysteine, 45.50, in ECL2) are Glu/Asp (e.g., GHRHR and PTH1R), ECL2 shows compact contacts with peptides through direct salt bridge (GHRHR: Arg^11P^ and D274^45.52b^), or intra-receptor salt bridge (PTH1R: K240^2.67b^ and D353^45.52b^). In the case of GCGR/GLP-1R whose residues at 45.52 are Thr, ECL2 of GCGR forms multiple hydrogen bonds with glucagon via Gly^4P^, Ser^8P^, and Ser^11P^, and ECL2 of GLP-1R forms polar interaction with multiple serines (Ser^14P^, Ser^17P^, and Ser^18P^) of GLP-1 via T298^45.52b^ and electrostatic interaction with His^7P^ via inward chain of R299^ECL2^. Remarkably, the binding of ExP5 induces reorganization of ECL2–peptide interface, where R299^ECL2^ rotates outward and forms salt bridges with Glu^16P^.

### G protein coupling by GHRHR

Like other class B GPCRs, the outward movement of the cytoplasmic end of TM6, and concomitant shift of TM5 and ICL3 of GHRHR form a cavity to accommodate α5 helix of Gα_s_. This process also involves TM2, TM3, ICL2, and helix 8. The interface residues in this cavity are highly conserved among class B GPCRs, and the arrangement of GHRHR–G_s_ complex is also similar to other class B GPCR–G_s_ complexes, and follows a common mechanism of G protein coupling. The high-resolution cryo-EM map allows us unambiguously to assign the water molecules between the interface of GHRHR and Gα_s_. Comparing with other class B GPCR–G_s_ complexes in which α5 helix of Gα_s_ loosely interacts with the receptor TM7–H8 hinge (Supplementary Fig. 9a, b), we found two water molecules in the GHRH–GHRHR–G_s_ complex establishing extensive polar interaction network connecting the C-terminus of Gα_s_ to the TM7–H8 hinge of GHRHR, thereby causing considerable conformational changes compared to GLP-1R–G_s_ and PTH1R–G_s_ structures (Supplementary Fig. 9c, d). Specifically, the crevice between the TM7–H8 hinge and TM6 in GHRHR–G_s_ complex is broader than that in the PTH1R–G_s_ and GLP-1R–G_s_ complexes, which may be responsible for G protein moving 3 Å toward the cytoplasmic core of the TMD bundle (Supplementary Fig. 9a, b).

Besides contacting the cytoplasmic cavity of TMs 2, 3, 6, and 7 induced by the opening of the cytoplasmic half of TM6 via Gα_s_, heterotrimeric G_s_ protein also establishes an electrostatic interaction network with ICL1 and H8 via Gβ (Supplementary Fig. 9e). Specifically, four residues (D312 of Gβ, R156^12.49b^ of ICL1, and E386^8.53b^ and R389^8.56b^ of H8) are clustered at the interface of GHRHR–Gβ by forming salt bridges. Such an organization is conserved across class B GPCRs, evidenced by the highly conserved residues and previous studies on GLP-1R and PTH1R. Notably, an additional component, Arg/Lys^8.60b^, from GLP-1R, GCGR, and PTH1R may join and further stabilizes the network, while G393^8.60b^ of GHRHR has neglectable contacts with Gβ. Indeed, G393^8.60b^R enhances the potency of GHRH, probably by strengthening the electrostatic interactions between GHRHR and G_s_, whereas the diminished potency of a double mutant (R156^12.49b^A/R389^8.56b^A) is likely resulted from the disruption of the electrostatic interaction network (Supplementary Fig. 9f).

### Implication of disease-causing mutations

**Fig. 4:**
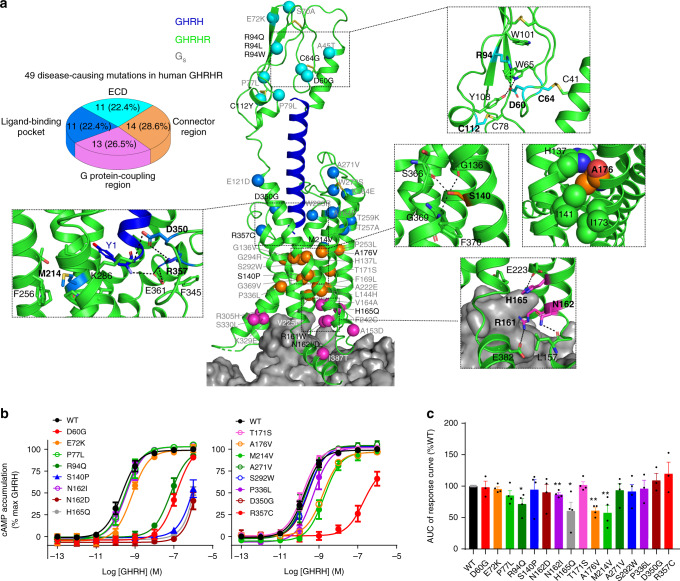
Disease-causing missense mutations of GHRHR. IGHD refers to conditions of growth hormone deficiency that are not necessarily associated with other pituitary hormone deficiencies or with an organic lesion. While there is no single cause identified for IGHD, defects in growth hormone, GHRHR and GH1 genes are implicated as causative factors^55^. Of which, naturally occurring missense mutations of GHRHR have been studied extensively^56^. **a** Naturally occurring mutations in the ECD, ligand-binding pocket, and G-protein-coupling region are colored cyan, marine, and magenta, respectively. The central region connecting the ligand-binding pocket and G-protein-coupling region is colored orange. **b**, **c** Functional assessment of effects of disease-causing GHRHR mutations on G_s_-mediated cAMP accumulation (**b**) and β-arrestin2 recruitment (**c**), cAMP accumulation assays and β-arrestin2 recruitment were performed in HEK 293 T cells and data shown are means ± S.E.M. of at least three independent experiments (*n* = 3–8), performed in quadruplicate or duplicate, respectively. Statistically significant differences were determined with a two-tailed Student’s *t* test. *P < 0.05, **P < 0.01 vs. wild type (WT); AUC area-under-the-curve. Source data are provided as a Source data file.

Based on the structure information, 25 missense mutants (21 were reported to be linked with IGHD previously; Fig. 4a and Supplementary Table 5) were made and assessed for their effects on cAMP signaling and β-arrestin2 recruitment. Four of them were further analyzed by MD simulations. It is established that a common fold of three-layer α-β-β/α architecture^30,31^ across class B GPCRs is stabilized by three conserved interlayer disulfide bonds (C41‒C64, C55‒C96, and C78‒C112) and the salt bridge/pi stacking within the family-wide conserved D-W-R/K-W motif (D60-W65-R94-W101) in GHRHR, that connect two β-sheets to directly interact with the peptide ligands. Presumably, naturally occurring mutations may disrupt the disulfide bonds (C64G and C112Y) or the conserved D-W-R/K-W motif (R94Q/L/W), thereby destabilizing the stability of ECD and preventing GHRH recognition. Indeed, our MD simulation and functional studies suggest that the IGHD-associated mutation R94Q^32^ breaks the salt bridge with D60, increases the flexibility of the ECD, decreases the area of GHRH–GHRHR interface, and reduces GHRH-induced cAMP accumulation (Fig. 4b and Supplementary Fig. 10a). We then examined if mutations on D60 could also eliminate the electrostatic interaction between D60 and R94 to exhibit similar functional and phenotypical outcomes. D60G is known for the little mouse phenomenon—the first naturally occurring animal model of inherited autosomal recessive GH-deficient dwarfism^33^. Like R94Q, it reduces GHRH binding affinity^34,35^, diminishes its potency on cAMP accumulation (Fig. 4b), and weakens GHRH binding in MD simulations (Supplementary Fig. 10a).

In the peptide-binding pocket, R357C was shown to loosen the compact GHRHR contacts in MD simulation (Supplementary Fig. 10b) and reduce GHRH potency by 1000-fold (Fig. 4b), consistent with the decreased potency of R357A and D350A mutants (Fig. 2d). Different from Ala/Val/Val in GLP-1R/PTH1R/GCGR, the residue at 3.41 of GHRHR located at the bottom of the ligand-binding pocket is M214, whose long side chain points to a hydrophobic cleft between TM4 and TM5. Another mutation associated with IGHD, M214V, was found to decrease GHRH potency by tenfold and selectively reduce β-arrestin2 recruitment (Fig. 4b).

The connector region is known for the sharp kink in the middle of TM6 around Pro^6.47b^-X-X-Gly^6.50b^. It appears that P336^6.47b^L hampered the sharp kink upon receptor activation evidenced by a reduced EC_50_ value for cAMP signaling (Fig. 4b). As a comparison, S140^1.50b^P eliminated the hydrogen bonds between TM1 and TM7, increased the flexibility of TMD region and the bound GHRH, and reduced cAMP accumulation by over 7000-fold (Fig. 4b and Supplementary Fig. 10b). A176^2.61b^ oriented toward TM1, and contributed close hydrophobic contacts with H137^1.47b^, I141^1.51b^, and I173^2.58b^. Substitution of A176^2.61b^ with a larger hydrophobic valine at this position reduced GHRH potency (tenfold) and β-arrestin2 recruitment (39%).

Within the G-protein-coupling region, N162^2.47b^I, N162^2.47b^D, and H165^2.50b^Q were previously proposed to be deleterious^36^—a view that was verified experimentally in this study (Fig. 4b) As shown in the structure (Fig. 4a), N162 formed a hydrogen bond with the backbone of L157^ICL1^, H165^2.50b^ from HETX motif^10,14^ played a crucial role in receptor activation, while H165Q might have directly altered receptor–G protein interface and abolished G protein coupling.

## Discussion

In summary, the high-resolution structure of the GHRHR–G_s_ complex, and mutational studies provide a basis for GHRH recognition and receptor activation. These results also unveiled three distinct malfunctioning mechanisms of GHRHR signaling: (i) impaired peptide binding in the ECD (D60G and R94Q); (ii) reduced ligand recognition (R357C)/G protein coupling (N162I/D and H165Q via functional validation); and (iii) disrupted signal propagation in the connector region (S140P). Together, our work solved a longstanding puzzle of IGHD-causing mutations, which result in the impairment of GHRHR-mediated signaling at the ECD or TMD, singly or in combination.

## Methods

### Cell culture

*Spodoptera frugiperda* (*Sf*9) insect cells (Expression Systems) were grown in ESF 921 serum-free medium (Expression Systems) at 27 °C and 120 r.p.m. HEK 293 T cells were purchased from the Cell Bank at the Chinese Academy of Sciences, cultured in Dulbecco’s modified Eagle’s medium (DMEM; Life Technologies) supplemented with 10% fetal bovine serum (Gibco) and maintained in a humidified chamber with 5% CO_2_ at 37 °C.

### Constructs of GHRHR and G_s_ heterotrimer

For structural studies, wild-type (WT) human GHRHR DNA (Genewiz) was cloned into pFastBac vector (Invitrogen) with its native signal sequence (M1-G22) replaced by the hemagglutinin (HA) signal peptide. Eighteen amino acids (A406–C423) were truncated at the C-terminus and LgBiT subunit (Promega) was fused with a 15-amino acid polypeptide (GSSGGGGSGGGGSSG) linker at the C-terminus followed by a Tev protease cleavage site and a double maltose-binding protein (MBP) tag to facilitate expression and purification. A dominant-negative human Gα_s_ (DNGα_s_) was generated by site-directed mutagenesis as previously described to stabilize the interaction with the βγ subunits^11,37^. Rat Gβ1 with an N-terminal 10× His-tag was fused with a SmBiT^38^ (peptide 86, Promega) subunit by the 15-amino acid polypeptide linker at its C-terminus. Human DNGα_s_, rat Gβ1, and bovine Gγ2 were cloned into pFastBac vector, respectively. In addition to clone the constructs into the pBiT vector (Promega) for NanoBiT assay, they all contained an N-terminal Flag tag (DYKDDDD) proceeded by a HA signal sequence, and were cloned into the pcDNA3.1 vector (Invitrogen) for functional studies.

To obtain a GHRHR–G_s_ complex with good homogeneity and stability, the human GHRHR was modified by replacing the N-terminal native signal peptide with HA sequence. In addition, 18 residues (A406–C423) were truncated at the C-terminus, followed by an LgBiT subunit and a double MBP affinity tag to improve protein yield and stability (Supplementary Fig. 2a). These modifications did not affect ligand binding and receptor activation (Supplementary Fig. 2b, c and Supplementary Table 1). In addition, a DNGα_s_^11,37^, His10-Gβ1-peptide 86 and Gγ2 were co-expressed with GHRHR(23-405)-15AA-LgBiT-2MBP in insect cells. Formation of GHRHR–G_s_ complex on the membrane was stimulated with an excess amount of GHRH, and in the presence of Gα- and Gβ-binding nanobody 35 (Nb35)^39^ (Supplementary Fig. 2d–e).

### Expression and purification of GHRHR–G_s_ complex

GHRHR-15AA-LgBiT-2MBP or GHRHR-2MBP, DNGα_s_, His10-Gβ1-peptide 86, and Gγ2 recombinant baculoviruses were prepared using Bac-to-Bac Baculovirus Expression System (Invitrogen) severally. *Sf*9 insect cells were grown to a density of 3 × 10^6^ cells per mL and then coinfected with four separate viruses at a ratio of 1:3:3:3 for GHRHR, DNGα_s_, Gβ1, and Gγ2. Cells were harvested by centrifugation 48 h post infection and pellets were stored at −80 °C until use.

The cell pellets were thawed on ice and lysed in a buffer containing 20 mM HEPES, pH 7.5, 100 mM NaCl, 10% (v/v) glycerol, 10 mM MgCl_2_, 5 mM CaCl_2_, 1 mM MnCl_2_, 100 μM TCEP, and supplemented with EDTA-free protease inhibitor cocktail (Bimake) by dounce homogenization. The complex formation was initiated by addition of 10 μM GHRH (GL Biochem), 10 μg/mL Nb35, 25 mU/mL apyrase (NEB), and the lysate was incubated for 1.5 h at room temperature (RT). The membrane was further solubilized by 0.5% (w/v) lauryl maltose neopentyl glycol (LMNG; Anatrace) and 0.1% (w/v) cholesterol hemisuccinate (CHS; Anatrace) for 2 h at 4 °C. After centrifugation at 65,000 × *g* for 30 min, the supernatant was isolated and incubated with amylose resin (NEB) for 2 h at 4 °C. The resin was then collected by centrifugation at 600 × *g* for 10 min, loaded into a gravity flow column (Sangon Biotech), and first washed with five column volumes of buffer containing 20 mM HEPES, pH 7.5, 100 mM NaCl, 10% (v/v) glycerol, 5 mM MgCl_2_, 1 mM MnCl_2_, 25 μM TCEP, 1 μM GHRH, 0.1% (w/v) LMNG, and 0.02% (w/v) CHS, followed by washing with 15 column volumes of buffer containing 20 mM HEPES, pH 7.5, 100 mM NaCl, 10% (v/v) glycerol, 5 mM MgCl_2_, 1 mM MnCl_2_, 25 μM TCEP, 1 μM GHRH, 0.03% (w/v) LMNG, 0.01% (w/v) glyco-diosgenin (Anatrace), and 0.008% (w/v) CHS. The protein was then incubated overnight with His-tagged Tev protease (customer-made) on the column to remove the C-terminal 2MBP-tag in the buffer above. The flow through was collected next day and concentrated with a 100 kDa molecular weight cut-off concentrator (Millipore). Concentrated GHRH–GHRHR–G_s_–Nb35 complex was loaded onto a Superdex 200 increase 10/300 GL column (GE Healthcare) with running buffer containing 20 mM HEPES, pH 7.5, 100 mM NaCl, 10 mM MgCl_2_, 100 μM TCEP, 5 μM GHRH, and 0.001% digitonin (Anatrace). The fractions for monomeric complex were collected and concentrated to 20–30 mg/mL for EM examination.

### Expression and purification of Nb35

Nb35 with a C-terminal 6× His-tag was expressed in the periplasm of *Escherichia coli* BL21 (DE3) cells, extracted and purified by nickel affinity chromatography as previously described^36^. Eluted protein was concentrated using a 10 kDa molecular weight cut-off concentrator (Millipore) and loaded onto a HiLoad 16/600 Superdex 75 column (GE Healthcare), with running buffer containing 20 mM HEPES, pH 7.5, and 100 mM NaCl. The monomeric fractions were pooled and supplemented with 30% (v/v) glycerol. Purified Nb35 was finally flash frozen in liquid nitrogen and stored in −80 °C.

### Cryo-EM data acquisition and image processing

The purified GHRH–GHRHR–G_s_ complex (3.5 μL) at a concentration of 22 mg/mL was applied to glow-discharged holey carbon grids (Quantifoil R1.2/1.3, 200 mesh), and subsequently vitrified using a Vitrobot Mark IV (ThermoFisher Scientific). Cryo-EM images were collected on a Titan Krios equipped with a Gatan K2 Summit direct electron. The microscope was operated at 300 kV accelerating voltage, at a nominal magnification of 29,000× in counting mode, corresponding to a pixel size of 1.014 Å. In total, 3813 image stacks were obtained at the dose rate of ~7.8 electrons per Å^2^ per second with a defocus range of −0.5 to −2.5 μm. The total exposure time was set to 8 s with intermediate frames recorded every 0.2 s, resulting in an accumulated of dose of 62 electrons per Å^2^.

Dose-fractionated image stacks were subjected to beam-induced motion correction and dose-weighting using MotionCor2.1 (ref. ^40^). A sum of all frames, filtered according to the exposure dose, in each image stack was used for further processing. Contrast transfer function parameters for each micrograph were determined by Gctf v1.06 (ref. ^41^). Further data processing was performed in RELION-3.0-beta2 (ref. ^42^). Particle selection, two-dimensional classification, and the first round of three-dimensional classification were performed on a binned dataset with a pixel size of 2.028 Å.

Auto-picking yielded 2,586,606 particle projections that were subjected to reference-free two-dimensional classification to discard false-positive particles or particles categorized in poorly defined classes, producing 1,456,108 projections for further processing. This subset of particle projections was subjected to consecutive rounds of three-dimensional classification with a pixel size of 2.028 Å. The map of PTH1R–G_s_ complex (EMDB-0410) low-pass filtered to 40 Å was used as an initial reference model for two rounds of three-dimensional classification, resulting in two subsets accounting for 481,220 projections that showed better EM densities. Further three-dimensional classifications focusing on the complex, with the exception of AHD of Gαs, produced one good subset with higher resolution, which was subsequently subjected to three-dimensional refinement and Bayesian polishing. The final refinement with frames 1–25 generated a map with an indicated global resolution of 2.6 Å, with 307,018 projections at a Fourier shell correlation of 0.143. Local resolution was determined using the Bsoft package with half maps as input maps^43^.

### Model building and refinement

The structure of the LA-PTH–PTH1R–G_s_ complex was used as an initial template for model building. Lipid coordinates and geometry restraints were generated using phenix.elbow. Models were docked into the EM density map using UCSF Chimera^44^. This starting model was then subjected to iterative rounds of manual adjustment and automated refinement in Coot^45^ and Phenix^46^, respectively. The final refinement statistics were validated using the module “comprehensive validation (cryo-EM)” in PHENIX. Structural figures were prepared in Chimera, Chimera X, and PyMOL (https://pymol.org/2/). The final refinement statistics are provided in Supplementary Table 2.

### NanoBiT assay

HEK 293 T cells were transiently transfected with GHRHR-LgBiT, Gα_s_, Gβ1-SmBiT (peptide 86 or 114), and Gγ2 48 h before assaying in a mass ratio of 1:1:1:1. Twenty-four hour post transfection, cells were digested and seeded into 96-well white plate (PerkinElmer) at optimal density (30,000 cells/well) and the medium was replaced with Opti-MEM (Gibco) 3 h prior to assaying. After equilibration to ambient temperature, the Nano-Glo Live Cell Substrate (Promega) was 20-fold diluted to create a 5× stock and mixed with cell culture medium to a final 1× concentration, then luminescence signal was measured using an EnVision Multimode Plate Reader (PerkinElmer). Following the baseline reading at 10 s interval for 2.5 min, GHRH was diluted with Opti-MEM and added to a final concentration of 10 nM, and reading continued at 10 s intervals for 10 min. RLU time-course response curves were normalized to that of background and calculated as percentage of the baseline value. Interaction intensity of the complex was expressed as area-under-the-curve (AUC) across the full kinetic trace (0–10 min).

### Negative-stain EM

Uranyl formate (0.75% (w/v), Electron Microscopy Sciences) solution was prepared as previously described^47^. Copper grids (300 mesh) coated with carbon film (Electron Microscopy Sciences) were glow charged, using PELCO easiGlow^TM^ Glow Discharge Cleaning System (Ted Pella Inc.) for 25 s at 25 mA. Purified protein samples (4 μL, 0.01 mg/mL) were applied to glow-charged holey grids for 1 min and then blotted off using filter paper. Uranyl formate solution (0.75%, 5 μL) was added to the grid surface twice: the first application was blotted off immediately and the second was retained for 1 min before blotting with filter paper. The stained girds were loaded into a Tecnai G2 Spirit transmission electron microscope (Thermo FEI) operated at 120 kV. Negative staining images were acquired at a magnification of 57,000× within a −1.5 to −2.0 μm defocus range.

### Dynamic light scattering

Dynamic light scattering determines size and size distribution by measuring the rapid changes in laser light intensity of molecules or particles in solution. Briefly, freshly purified protein samples (15 μL each) were concentrated to 1 mg/mL and loaded into DynaPro NanoStar (Wyatt Technology) to measure time-dependent fluctuations of scattered intensity at 30 °C. Data were analyzed using the dynamics software supplied with the instrument.

### cAMP accumulation assay

GHRH-stimulated cAMP accumulation was measured by a LANCE Ultra cAMP kit (PerkinElmer). Briefly, 24 h after transfection with GHRHR(23-423), GHRHR(23-405), GHRHR(23-405)-LgBiT-2MBP, or HA-Flag-GHRHR (WT and mutants), HEK 293 T cells (3000/well in 384-well white plates, PerkinElmer) were digested by 0.2% (w/v) EDTA and 5 μL stimulation buffer (HBSS supplemented with 5 mM HEPES, 0.5 mM IBMX, and 0.1% (w/v) BSA, pH 7.4). Different concentrations (5 μL) of GHRH were then added and the stimulation lasted for 30 min at RT. The reaction was stopped by adding 5 μL Eu-cAMP tracer and 5 μL ULight-anti-cAMP. After 1 h RT incubation, TR-FRET signals (excitation: 320 nm, emission: 615 and 665 nm) were measured by an EnVision (PerkinElmer).

### Whole cell binding assay

This assay was conducted in 96-well Iso-plates (PerkinElmer) coated with poly-D-lysine hydrobromide (Sigma-Aldrich). Twenty-four hour after transfection with GHRHR(23-423) or GHRHR(23-405)-LgBiT-2MBP, HEK 293 T cells were washed twice, and incubated with blocking buffer (DMEM medium supplemented with 33 mM HEPES, and 0.1% (w/v) BSA, pH 7.4) for 2 h at 37 °C. Then, radiolabeled ^125^I-GHRH (80 pM, Phoenix Biotech) and seven decreasing concentrations of unlabeled peptide (10 μM, five serial gradient dilutions) were added and competitively reacted with the cells in binding buffer (PBS supplemented with 10% (w/v) BSA, pH 7.4) at RT for 3 h. Cells were washed with ice-cold PBS and lysed by 50 μL lysis buffer (PBS supplemented with 20 mM Tris-HCl and 1% (v/v) Triton X-100, pH 7.4). Finally, 150 μL of scintillation cocktail (OptiPhase SuperMix, PerkinElmer) was employed and radioactivity (counts per minute) read in a scintillation counter (MicroBeta^2^ plate counter, PerkinElmer).

### β-arrestin2 recruitment assay

HEK 293 T cells were seeded at a density of 30,000 cells per well into 96-well culture plates pretreated with poly-D-lysine hydrobromide. After incubation for 24 h to reach 70–80% confluence, the cells were transiently transfected with HA-Flag-GHRHR-Rluc8 (WT and mutants) and β-arrestin2-Venus at a 1:9 mass ratio, using Lipofectamine 3000 reagent (Invitrogen) and cultured for another 24 h. Thereafter, cells were washed once and incubated for 30 min at 37 °C with HBSS buffer (pH 7.4) supplemented with 0.1% (m/v) BSA and 10 mM HEPES. A total of 5 μM coelenterazine h (YEASEN Biotechnology) was then added and incubated for 5 min in the dark. The bioluminescence resonance energy transfer (BRET) signals were detected with an EnVision by calculating the ratio of emission at 535 nm over emission at 470 nm. A 1.5 min baseline of BRET measurements was taken before the addition of 250 μM GHRH or vehicle and BRET signal was measured at 10 s intervals for further 9 min. After removing baseline and background readings by subtracting average values of the baseline measurement and average values of vehicle-treated samples, respectively, the AUC across the time-course response curve was determined and normalized to the WT, which was set to 100%.

### Molecular dynamics simulation

All-atom MD simulations of the GHRH–GHRHR were performed by Gromacs 2018.5. After removing all G protein subunits and heteroatoms, the receptor and its disease-causing mutants (D60G, R94Q, S140P, and R357C) were prepared and capped by the Protein Preparation Wizard (Schrodinger 2017-4). Titratable residues were left in their dominant state at pH 7.0. The complexes were embedded in a bilayer composed of 225 POPC lipids and solvated with 0.15 M NaCl in explicitly represented waters, using CHARMM-GUI Membrane Builder^48^. The CHARMM36-CAMP force filed^49^ was adopted for protein, peptides, lipids, and salt ions, while the CHARMM TIP3P model was chosen for water. The particle mesh Ewald method^50^ was used to treat all electrostatic interactions beyond a cutoff of 10 Å and the bonds involving hydrogen atoms were constrained using LINCS algorithm^51^. The constructed system was firstly relaxed using the steepest descent energy minimization, followed with slow heating of the system to 310 K with restraints. The restraints were reduced gradually over 50 ns, with a simulation step of 1 fs. Finally, a 1 μs production run without restraints was carried out for each simulation, with a time step of 2 fs in the NPT ensemble at 310 K and 1 bar using the v-rescale thermostat^52^ and the semiisotropic Parrinello–Rahman barostat^53^, respectively. The last 500 ns trajectory was used for analysis, and the GHRH–GHRHR interface area was calculated by the program FreeSASA^54^, using the Sharke-Rupley algorithm with a probe radius of 1.2 Å.

### Statistical analysis

All functional study data were analyzed using Prism 7 (GraphPad) and presented as means ± S.E.M. from at least three independent experiments. Concentration–response curves were evaluated with a three-parameter logistic equation. The significance was determined with either two-tailed Student’s *t* test or one-way ANOVA, and *P* < 0.05 was considered statistically significant.

### Reporting summary

Further information on research design is available in the Nature Research Reporting Summary linked to this article.

## Supplementary information

Supplementary Information

Reporting Summary

## Data Availability

Data supporting the findings of this manuscript are available from the corresponding authors upon reasonable request. A reporting summary for this article is available as a Supplementary information file. The atomic coordinates and the electron microscopy maps have been deposited in the Protein Data Bank (PDB) under accession number PDB 7CZ5 and Electron Microscopy Data Bank (EMDB) accession number EMD-30505. Source data are provided with this paper.

## Source data

Source Data
