# Supplementary material for: Structural basis for activation of the growth hormone-releasing hormone receptor
Source: Nat Commun. 2020 Oct 15;11:5205. doi: 10.1038/s41467-020-18945-0 (PMC7567103; doi:10.1038/s41467-020-18945-0)
Supplement: Supplementary file 1 — Supplementary Information [file 41467_2020_18945_MOESM1_ESM.pdf]

Supplementary Information

Structural basis for activation of the growth hormone-releasing hormone receptor

Brief description of what this file includes:

Supplementary Fig 1. Stabilization of GHRHR–G_s complex with NanoBiT.

Supplementary Fig 2. Purification and characterization of the GHRH–GHRHR–G_s–Nb35 complex.

Supplementary Fig 3. Cryo-EM micrograph and 2D class averages of the GHRH–GHRHR–G_s complex.

Supplementary Fig 4. Single-particle cryo-EM analysis and resolution of the cryo-EM map.

Supplementary Fig 5. Atomic-resolution model of the GHRH–GHRHR–G_s complex in the cryo-EM density map.

Supplementary Fig 6. Sequence conservation analysis of GHRH and the peptide-binding pockets of GHRHR across diversified species.

Supplementary Fig 7. Functional role of the ECD.

Supplementary Fig 8. Comparison of peptide-binding modes among class B GPCRs.

Supplementary Fig 9. Structure comparison of GHRHR–G_s interface with that of other class B GPCRs.

Supplementary Fig 10. Molecular dynamics (MD) simulation of GHRHR with disease-causing mutations.

Supplementary Table 1. Effects of GHRH-mediated cAMP accumulation.

Supplementary Table 2. Cryo-EM data collection, model refinement and validation statistics.

Supplementary Table 3. Interaction of GHRH N-terminal helix with TMD of GHRHR.

Supplementary Table 4. Effects of residue mutation in the ligand-binding pocket on GHRH-induced cAMP accumulation.

Supplementary Table 5. Summary of disease-causing missense mutations in GHRHR.

Supplementary Table 6. List of primers sequences for site-direct mutagenesis studies, related to Figs. 2, 4, 5, Supplementary Figs. 6, 8 and Supplementary Tables 4, 5.

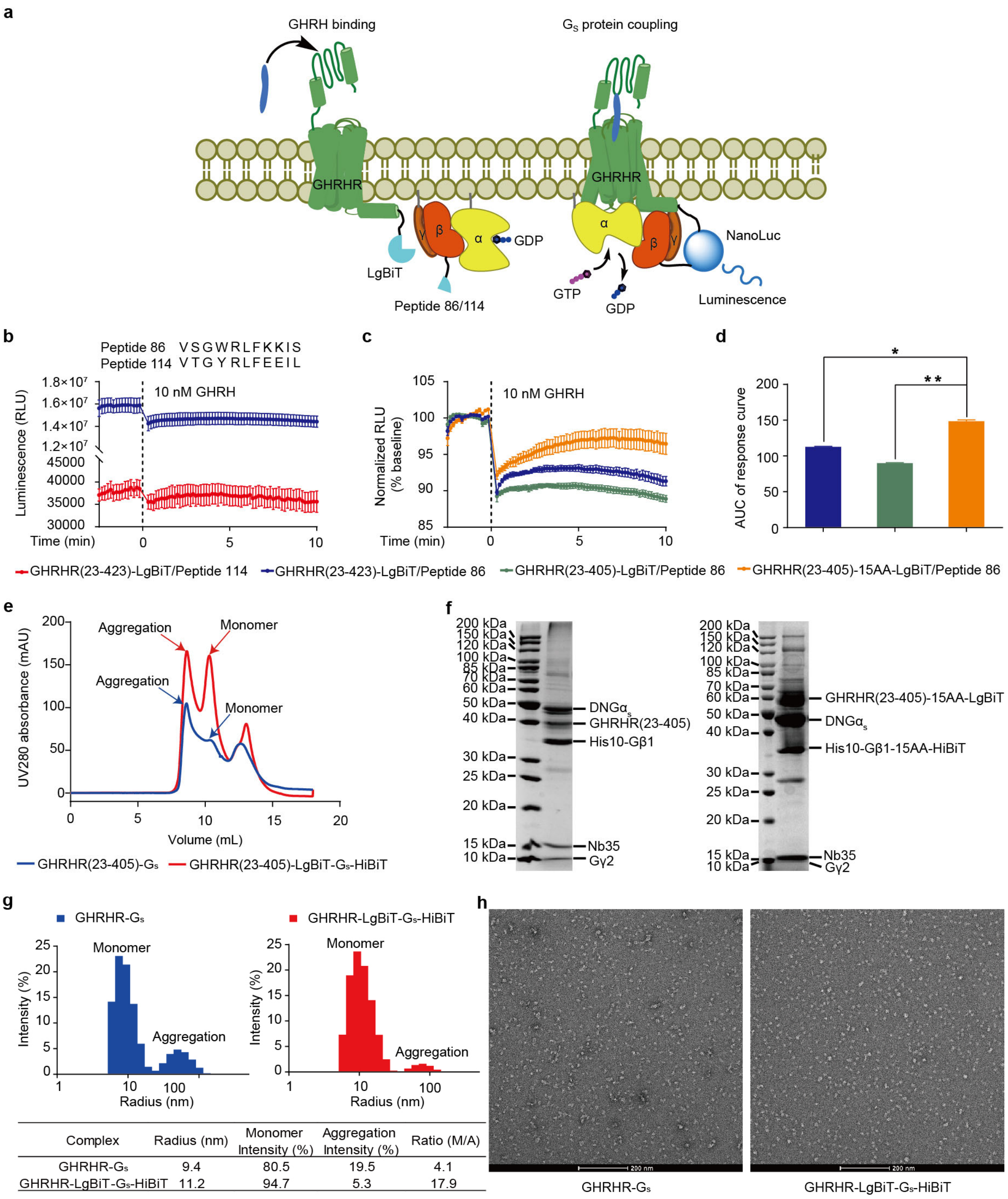

Complex	Radius (nm)	Monomer Intensity (%)	Aggregation Intensity (%)	Ratio (M/A)
GHRHR-Gs	9.4	80.5	19.5	4.1
GHRHR-LgBiT-Gs-HiBiT	11.2	94.7	5.3	17.9

Supplementary Figure 1. Stabilization of GHRHR–Gs complex with NanoBiT. NanoBiT assay was designed to investigate the protein-protein interactions (PPIs) based on protein-fragment complementation, and comprised of ~18 kDa LgBiT and ~1.3 kDa SmBiT (peptide 114, K_D = 190 μM) subunits which were split from the NanoLuc, a small luciferase with high physical stability and sensitivity[1, 2, 3]. In this assay system, PPIs would bring NanoBiT subunits (fused to the two interacted proteins, respectively) to close proximity forming a functional luciferase and giving a signal. The weak intrinsic affinity between LgBiT and peptide 114 will be dramatically increased when SmBiT is replaced by another variant, peptide 86 (K_D = 700 pM)[2]. Taking advantage of this attribute, we designed a strategy that could enhance the association of the GHRHR–Gs protein complex through the high intrinsic affinity of two subunits. By fusing peptide 86 to the C terminus of His10-

Gβ1 and LgBiT to that of the receptor with 15-amino acid linkers (Extended Data Fig. 1a), a stronger luminescence signal (Extended Data Fig. 1b) as well as a slower dissociation of receptor–G_s complex were observed, respectively, compared to peptide 114 and other linkers between the receptor and LgBiT (Extended Data Fig. 1c-d and Extended Data Table 1). The complex was therefore further stabilized by the interaction between LgBiT and SmBiT. **a**, Schematic of NanoBit subunits functioned during GHRH–GHRHR–G_s complex formation. **b**, Top panel, the amino acid sequence of peptides 86 and 114; bottom panel, effect of two SmBiT peptides on the kinetics of complex formation detected by luminescence signal. **c**, Effect of LgBiT position on the kinetics of complex formation detected by luminescence signal. The dashed line indicates the time point of 10 nM GHRHR addition. **d**, Comparison of peptides 86 and 114 for enhancing complex association, quantified by AUC (0-10 min) of RLU response curve and normalized to the baseline. **e**, Respective size-exclusion chromatography elution profiles of the GHRHR–G_s and GHRHR–LgBiT–G_s–HiBiT complexes. The peaks for aggregation and monomer are indicated. **f**, SDS-PAGE analysis of the purified complexes concentrated from the monomeric fractions by Coomassie blue staining. This experiment was repeated independently twice with similar results. **g**, Upper panel, dynamic light scattering (DLS) size distribution histogram of GHRHR–G_s and GHRHR–LgBiT–G_s–HiBiT complexes. Lower panel, values of radius, percentage of intensity for monomer and aggregation, and ratios of monomer/aggregation (M/A). **h,** Representative negative staining images of the corresponding complexes. This experiment was repeated independently twice with similar results. Scale bar is 200 nm. Data are displayed as means ± S.E.M. of at least three independent experiments performed in duplicate. Statistical significance was determined with a two-tailed Student's *t*-test; *P < 0.05, **P < 0.01; AUC, area-under-the-curve; RLU, relative luminometer unit. Effects of NanoBiT tethering strategy on the stability of GHRHR–G_s complex were investigated by gel filtration chromatography, SDS-PAGE, dynamic light scattering (DLS) and negative staining EM. It was found that using this approach, (i) the complex had a higher monomer peak distribution than that of wild-type (WT), indicative of an increased stability (**e**, **f**); (ii) monodispersity of complexes evaluated by DLS showed 4.3-fold increase in monomer (radius of ~10 nm)/aggregation (radius of ~100 nm) ratio compared to the WT (**g**); and (iii) improved integrity and homogeneity of complex particles were observed using negative staining (**h**). These results are consistent with previous studies on type 1 vasoactive intestinal polypeptide receptor (VIP1R) and CC chemokine receptor 7 (CCR7)[4]. Source data are provided as a Source Data file.

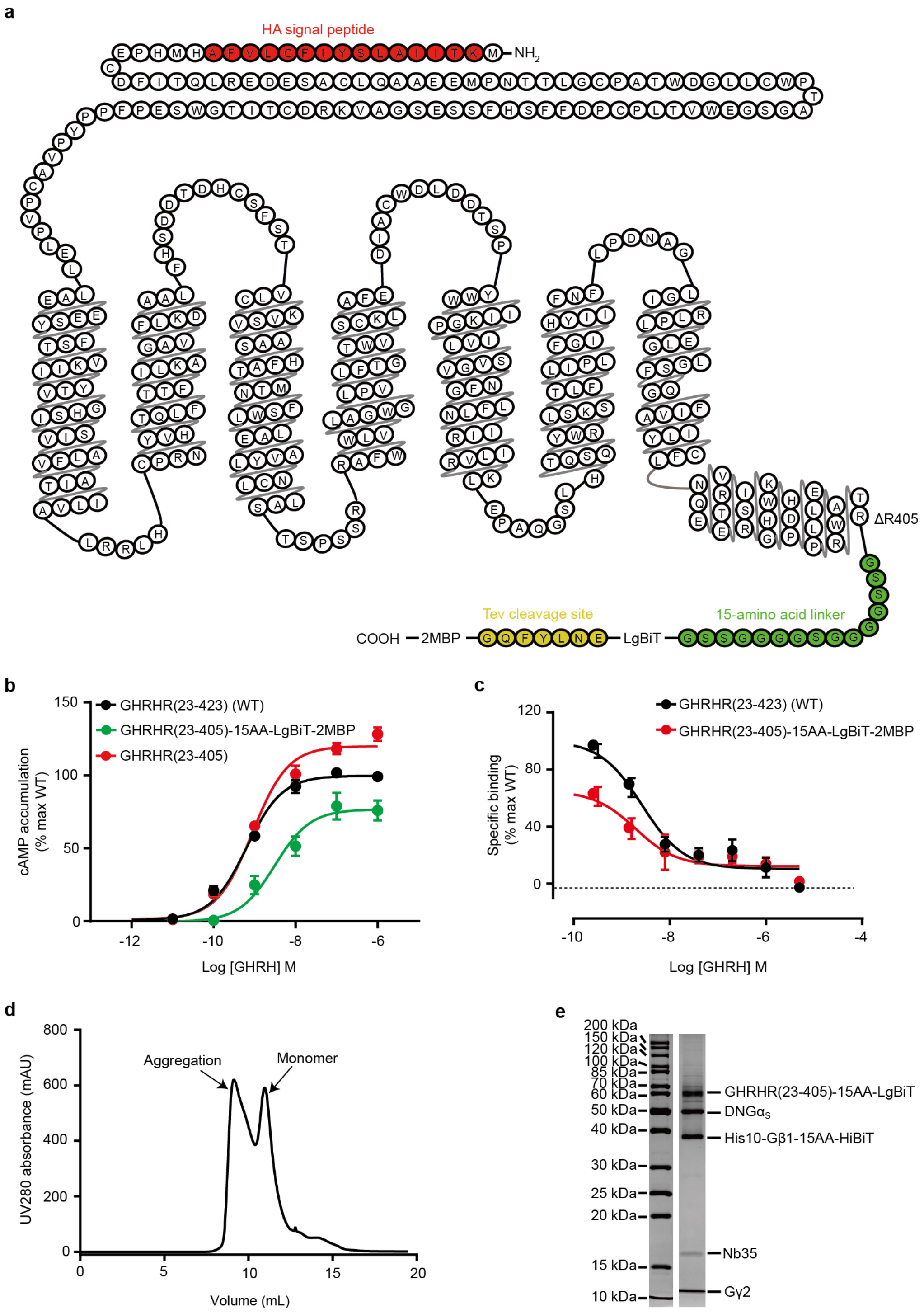

Supplementary Figure 2. Purification and characterization of the GHRH–GHRHR–G_s–Nb35 complex. a, Schematic of HA-GHRHR(23-405)-15AA-LgBiT-Tev-2MBP construct used in the study. The HA signal peptide (red), 15-amino acid (AA) linker (green), Tev cleavage site (yellow) and R405 truncation site are highlighted and indicated. **b**, cAMP concentration-response curves for GHRHR(23-423) (wild-type, WT, black), GHRHR(23-405) (red) and GHRHR(23-405)-15AA-LgBiT-2MBP (green). Concentration-response curves for cAMP accumulation of WT and mutant receptors were stimulated by GHRH in HEK 293T cells after 24 h

of transfection. Data are normalized by the WT receptor and fitted with a three-parameter logistic equation. All values are means ± S.E.M. of at least three independent experiments conducted in quadruplicate (n = 8-10). **c**, Competitive inhibition of ^{125}I-GHRH binding by GHRH. Binding affinity is quantified by reduction of radioactivity (counts per minute, CPM) and normalized to the maximal response of WT receptor. Binding affinity data are fitted with a three-parameter logistic equation. All values are means ± S.E.M. of at least three independent experiments (n = 7-8), conducted in duplicate. **d**, Representative size-exclusion chromatography elution profile of MBP-purified complex on Superdex 200 Increase 10/300 column. The peaks of aggregation and monomer are indicated by arrows. **e**, SDS-PAGE analysis of the purified complex concentrated from the monomeric fraction by Coomassie blue staining. This experiment was repeated independently twice with similar results. Source data are provided as a Source Data file.

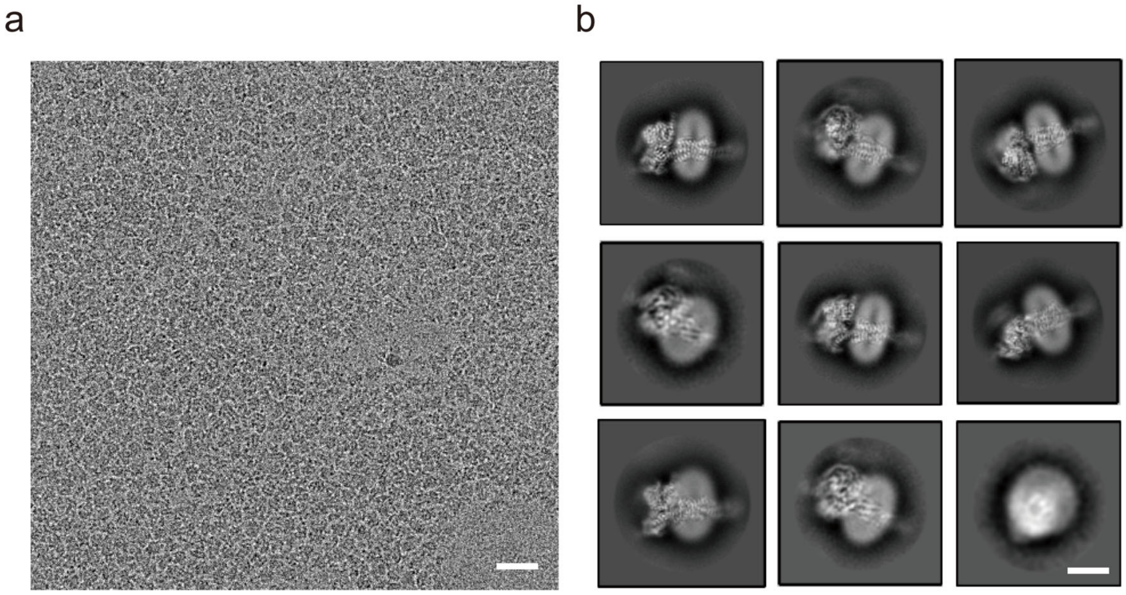

Supplementary Figure 3. Cryo-EM micrograph and 2D class averages of the GHRH–GHRHR–G_s complex. a, Representative cryo-EM micrograph of GHRH–GHRHR–G_s complex. Scale bar, 30 nm. **b**, Representative reference-free 2D class averages. Scale bar, 5 nm. 2D, two-dimensional. This experiment was repeated independently twice with similar results.

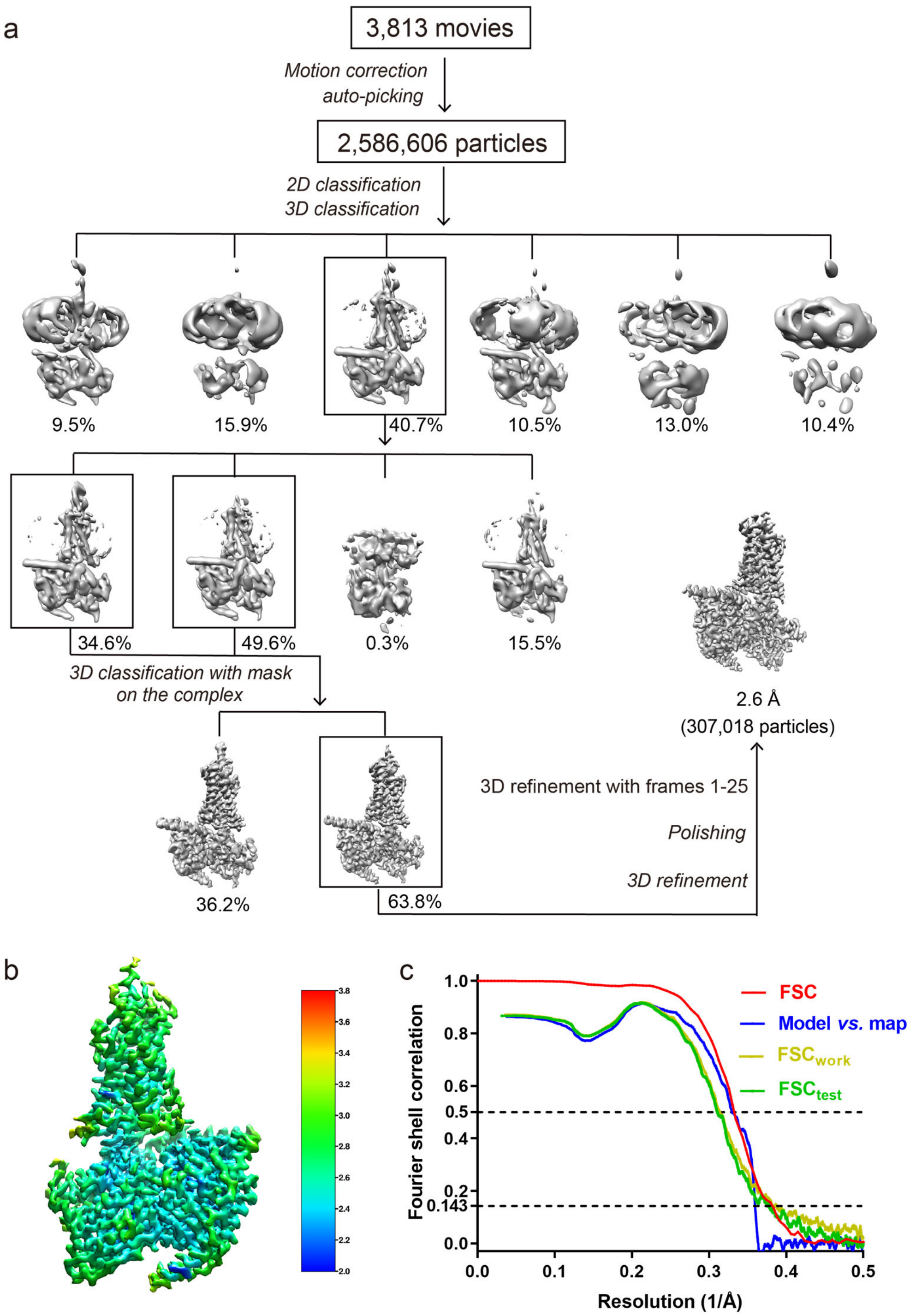

Supplementary Figure 4. Single-particle cryo-EM analysis and resolution of the cryo-EM map. a, Flow chart of cryo-EM data processing. **b**, 3D density map colored according to local resolution (Å). **c,** Gold-standard Fourier shell correlation curve (red), FSC of the refined model *vs*. the map curve (blue) and FSCwork/FSCtest validation curves (yellow and green, respectively). The resolutions at FSC=0.143 and FSC=0.5 are indicated with dashed lines.3D, three-dimensional.

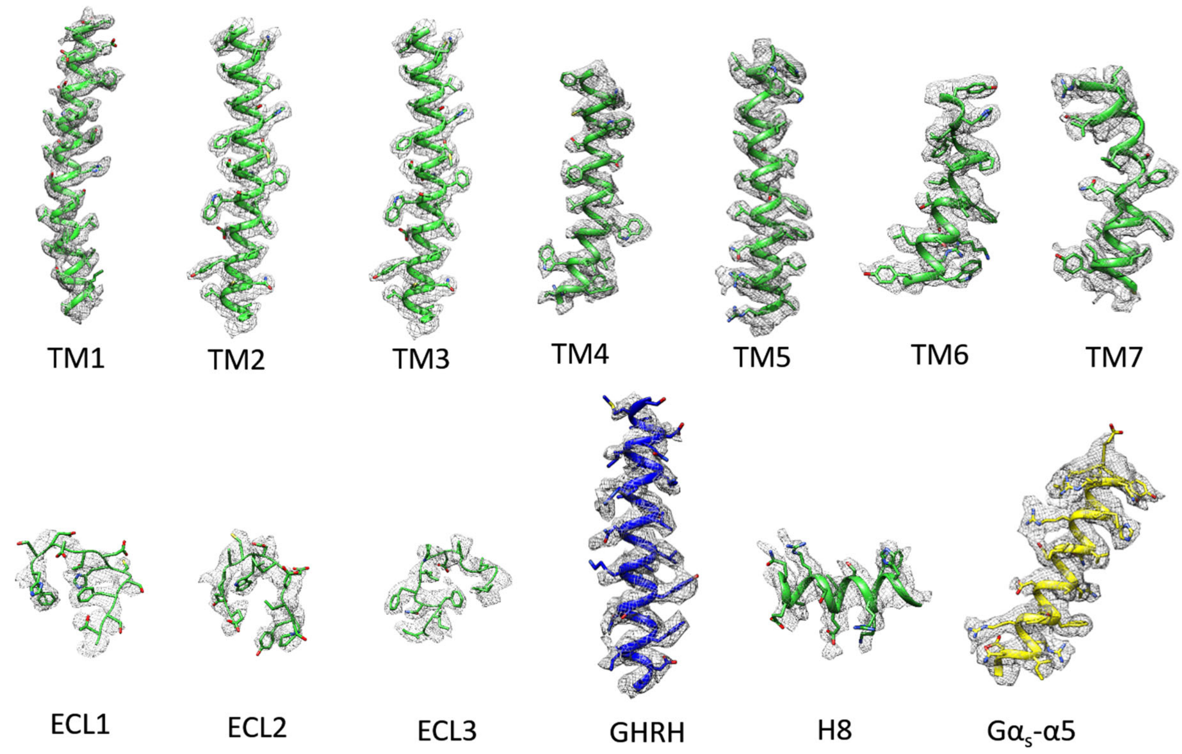

Supplementary Figure 5. Atomic-resolution model of the GHRH–GHRHR–G$_s$ complex in the cryo-EM density map. EM density map and model are shown for all seven-transmembrane α-helix, ECL1-3, and helix 8 of GHRHR, GHRH peptide and the α5-helix of the Gα$_s$ Ras-like domain. The densities of most residues are clearly seen in the map.

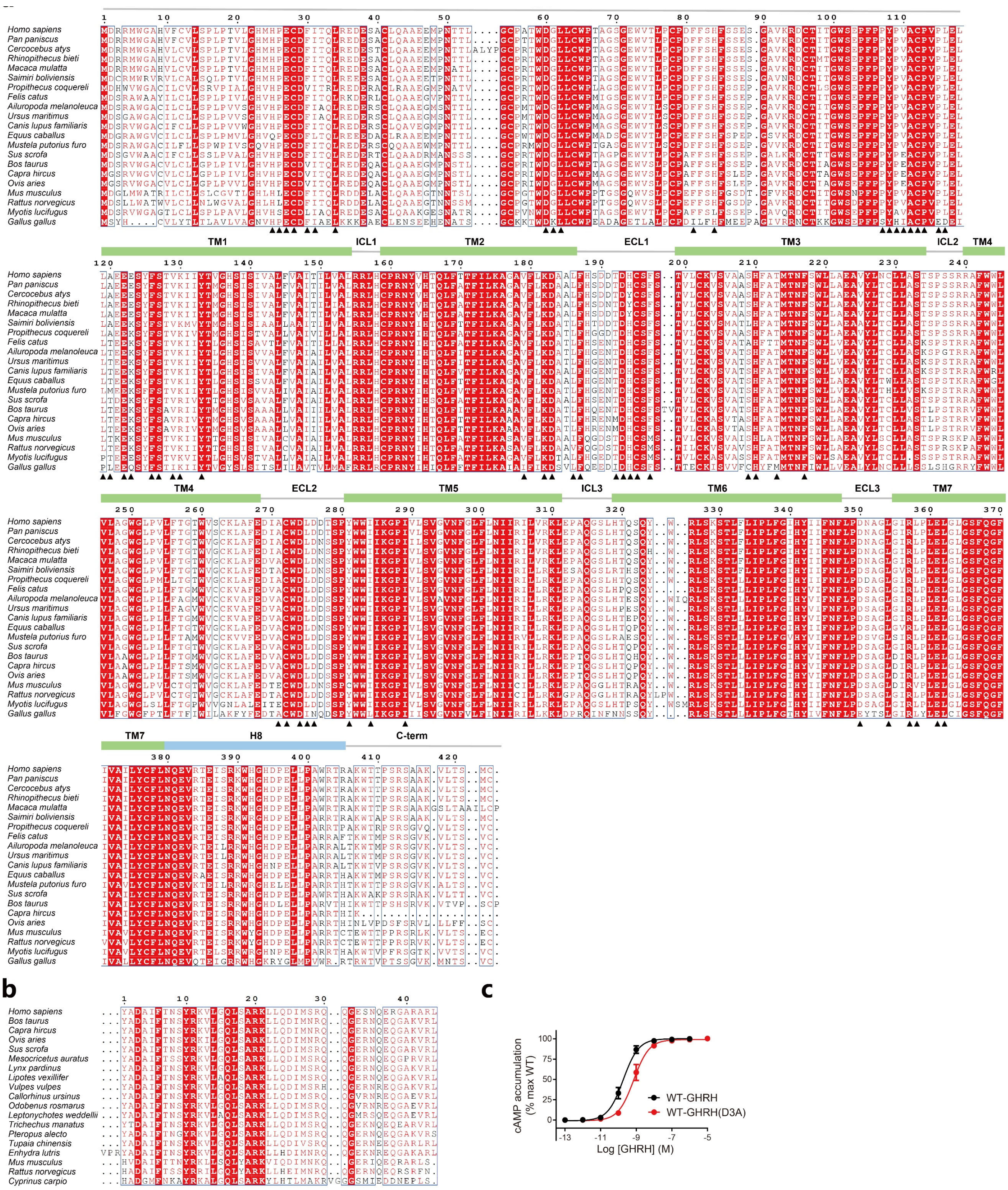

Supplementary Figure 6. Sequence conservation analysis of GHRH and the peptide-binding pockets of GHRHR across diversified species. a, Multiple sequence alignment of GHRHR in 21 species. Residues within 5Å of GHRH are highlighted by solid triangles. **b,** Multiple sequence alignment of GHRH in 19 species. **c,** Effect of GHRH mutant D3A on GHRHR-mediated cAMP accumulation. Data are presented as mean values ± S.E.M. of four independent experiments (n = 4), conducted in quadruplicate. Source data are provided as a Source Data file.

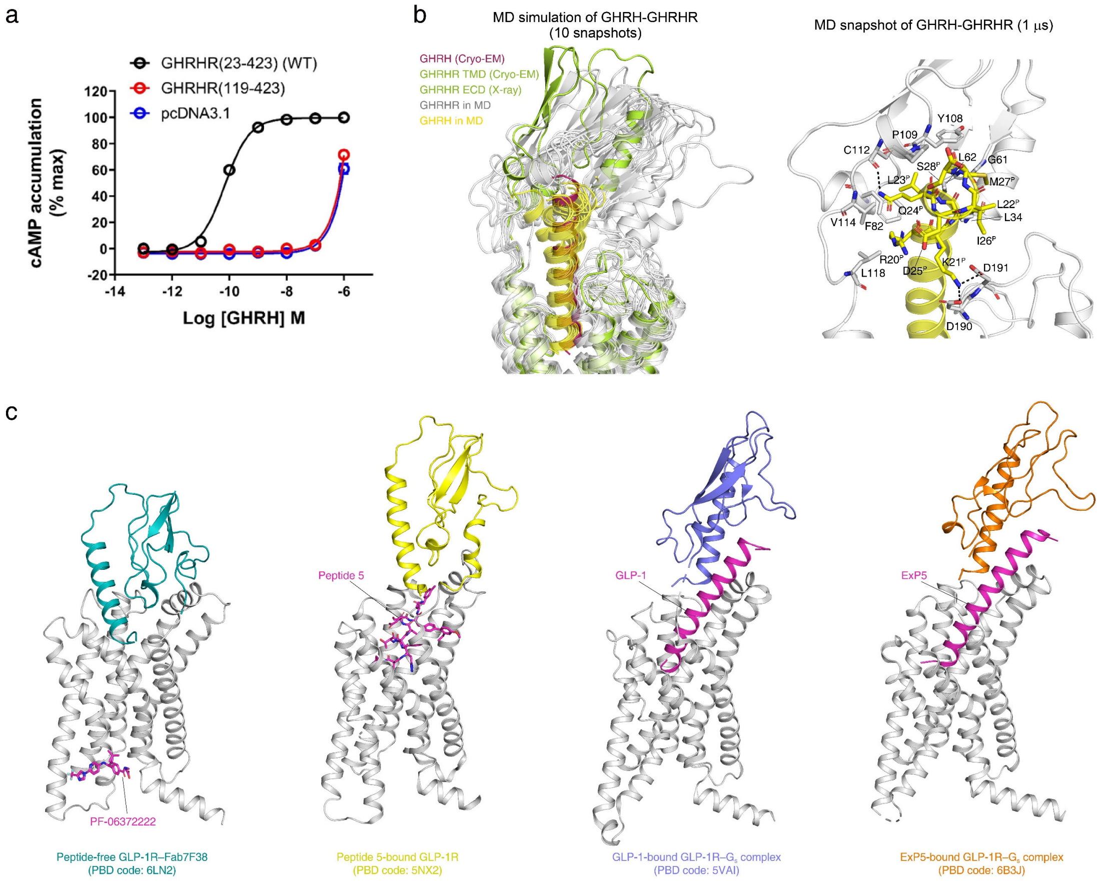

Supplementary Figure 7. Functional role of the ECD. a, Concentration-response curves of GHRH in activating cAMP signaling in wild-type (WT) and ECD-truncated human GHRHR expressing HEK 293T cells. Data shown are means ± S.E.M. of three independent experiments (n = 3), conducted in quadruplicate. **b**, MD simulation of GHRHR bound with GHRH. Left, 10 snapshots from 1 μs simulation trajectory were extracted to present the dynamic conformations of GHRHR ECD. Right, molecular recognition of the C-terminal region of GHRH by the ECD of GHRHR in the finial snapshot. **c,** Conformational comparison of GLP-1R ECD in different states[5-8]. G proteins are omitted for clarity. All structures are superimposed on the cryo-EM structure of the GLP-1–GLP-1R–G_s complex using the heavy atoms of residues A153$^{1.48b}$–S163$^{1.58b}$ (TM1), I179$^{2.49b}$–I196$^{2.66b}$ (TM2), V229$^{3.32b}$–G248$^{3.51b}$ (TM3), G273$^{4.49b}$–P277$^{4.53b}$ (TM4), T353$^{6.42b}$–I357$^{6.46b}$ (TM6), and Q394$^{7.45b}$–Y402$^{7.53b}$ (TM7). Source data are provided as a Source Data file.

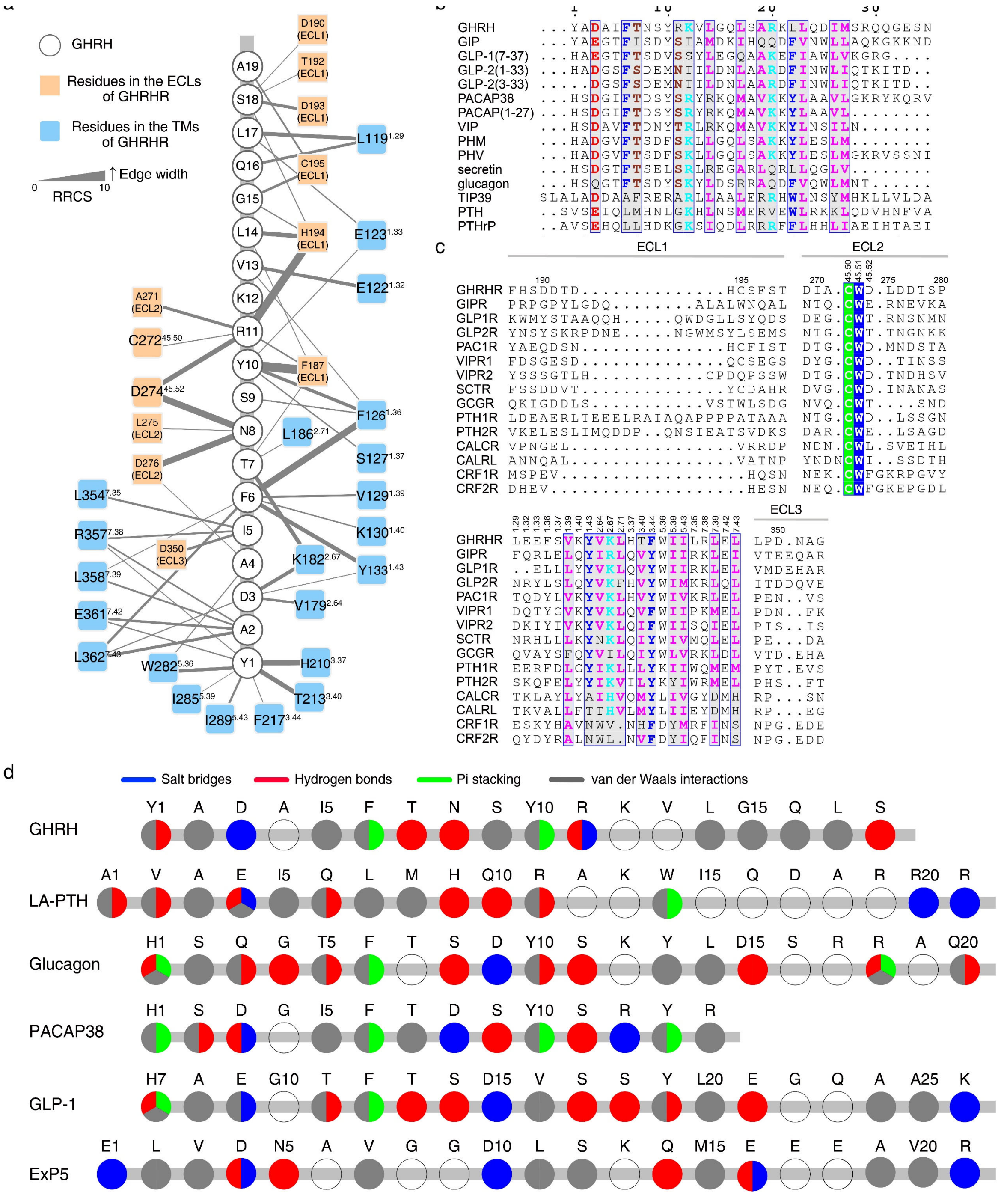

a
GHRH
Residues in the ECLs of GHRHR
Residues in the TMs of GHRHR
Edge width
RRCS 0 10
A19 S18 L17 Q16 G15 L14 V13 K12 R11 Y10 S9 N8 T7 F6 I5 A4 D3 A2 Y1
D190 (ECL1) T192 (ECL1) D193 (ECL1) C195 (ECL1) H194 (ECL1) F187 (ECL1)
A271 (ECL2) C272 45.50 D274 45.52 L275 (ECL2) D276 (ECL2) D350 (ECL3)
L119 1.29 E123 1.33 E122 1.32 F126 1.36 L186 2.71 S127 1.37 V129 1.39 K130 1.40 K182 2.67 Y133 1.43 V179 2.64 H210 3.37 T213 3.40 F217 3.44 I289 5.43 I285 5.39 W282 5.36
L354 7.35 R357 7.38 L358 7.39 E361 7.42 L362 7.43
b
1 10 20 30
GHRH ...YADAIFTNSYRKVLGQLSARKLLQDIMSRQQGESN
GIP ...YAEGTFISDYSIAMDKIHQQDFVNWLLAQKGKKND
GLP-1(7-37) ...HAEGTFTSDVSSYLEGQAAKEFIAWLVKGRG....
GLP-2(1-33) ...HADGSFSDEMNTILDNLAARDFINWLIQTKITD..
GLP-2(3-33) .....DGSFSDEMNTILDNLAARDFINWLIQTKITD..
PACAP38 ...HSDGIFTDSYSRYRKQMAVKKYLAAVLGKRYKQRV
PACAP(1-27) ...HSDGIFTDSYSRYRKQMAVKKYLAAVL........
VIP ...HSDAVFTDNYTRLRKQMAVKKYLNSILN.......
PHM ...HADGVFTSDFSKLLGQLSAKKYLESLM........
PHV ...HADGVFTSDFSKLLGQLSAKKYLESLMGKRVSSNI
secretin ...HSDGTFTSELSRLREGARLQRLLQGLV........
glucagon ...HSQGTFTSDYSKYLDSRRAQDFVQWLMNT......
TIP39 SLALADDAAFRERARLLAALERRHWLNSYMHKLLVLDA
PTH ..SVSEIQLMHNLGKHLNSMERVEWLRKKLQDVHNFVA
PTHrP ..AVSEHQLLHDKGKSIQDLRRRFFLHHLIAEIHTAEI
c
ECL1 ECL2
190 195 270 45.50 45.51 45.52 275 280
GHRHR FHSDDTD..............HCSFST DIA.CWD.LDDTSP
GIPR PRPGPYLGDQ.......ALALWNQAL NTQ.CWE.RNEVKA
GLP1R KWMYSTAAQQH.....QWDGLLSYQDS DEG.CWT.RNSNMN
GLP2R YNSYSKRPDNE.....NGWMSYLSEMS NTG.CWT.TNGNKK
PAC1R YAEQDSN..............HCFIST DTG.CWD.MNDSTA
VIPR1 FDSGESD..............QCSEGS DYG.CWD..TINSS
VIPR2 YSSSGTLH...........CPDQPSSW DTG.CWD.TNDHSV
SCTR FSSDDVT..............YCDAHR DVG.CWD.INANAS
GCGR QKIGDDLS...........VSTWLSDG NVQ.CWT....SND
PTH1R LDEAERLTEEELRAIAQAPPPPATAAA NTG.CWD..LSSGN
PTH2R VKELESLIMQDDP..QNSIEATSVDKS DAR.CWE..LSAGD
CALCR VPNGEL................VRRDP NDN.CWL.SVETHL
CALRL ANNQAL................VATNP YNDNCWI..SSDTH
CRF1R MSPEV..................HQSN NEK.CWFGKRPGVY
CRF2R DHEV...................HESN NEQ.CWFGKEPGDL
ECL3
1.29 1.32 1.33 1.36 1.37 1.39 1.40 1.43 2.64 2.67 2.71 3.37 3.40 3.44 5.36 5.39 5.43 7.35 7.38 7.39 7.42 7.43 350
GHRHR LEEFSVKYVKLHTFWIILRLEL LPD.NAG
GIPR FQRLELQYIRLQVYWIIRKLEI VTEEQAR
GLP1R ..ELLLYYVKLQVYWIIRKLEL VMDEHAR
GLP2R NRYLSLQYVKFHVYWIMKRLQL ITDDQVE
PAC1R TQDYLVKYVKLHVYWIVKRLEL PEN..VS
VIPR1 DQTYGVKYVKLQVFWIIPKMEL PDN..FK
VIPR2 DKIYIVKYVKLQIFWIISQIEL PIS..IS
SCTR NRHLLLKYNKLQIYWIVMQLEL PE...DA
GCGR QVAYSFQYVILQIYWLVRKLDL VTD.EHA
PTH1R EERFDLGYIKLLLYKIIWQMEM PYT.EVS
PTH2R SKQFELYYIKVILYKYIWRMEL PHS..FT
CALCR TKLAYLYAIHVQMYLIVGYDMH RP...SN
CALRL TKVALLFTTHVLMYLIIEYDMH RP...EG
CRF1R ESKYHAVNWV.NHFDYMRFINS NPG.EDE
CRF2R QYDYRALNWL.NVFDYIQFINS NPG.EDD
d
Salt bridges
Hydrogen bonds
Pi stacking
van der Waals interactions
GHRH Y1 A D A I5 F T N S Y10 R K V L G15 Q L S
LA-PTH A1 V A E I5 Q L M H Q10 R A K W I15 Q D A R R20 R
Glucagon H1 S Q G T5 F T S D Y10 S K Y L D15 S R R A Q20
PACAP38 H1 S D G I5 F T D S Y10 S R Y R
GLP-1 H7 A E G10 T F T S D15 V S S Y L20 E G Q A A25 K
ExP5 E1 L V D N5 A V G G D10 L S K Q M15 E E E A V20 R

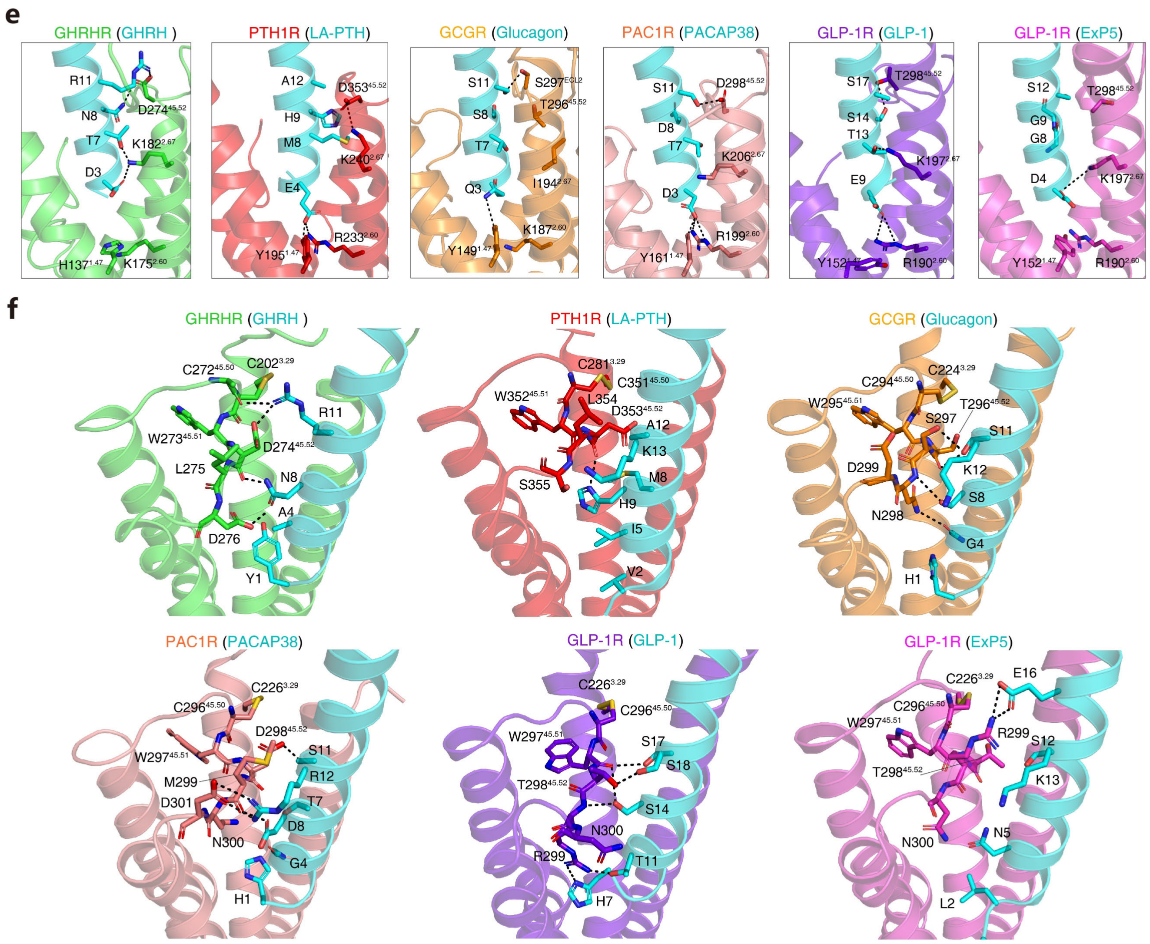

Supplementary Figure 8. Comparison of peptide-binding modes among class B GPCRs. a, Packing interactions between GHRH and GHRHR, with the line thickness representing the residue-residue contact core (RRCS)[9]. **b,** Sequence alignment of class B GPCR peptide hormones, residue numbers are shown based on GHRH. **c,** Sequence alignment in the peptide-binding pockets among 15 class B GPCRs. Residue numbers are shown based on GHRHR, with class B GPCR numbers[10, 11] on the top. The complementary residue interactions between GHRH and GHRHR (Tyr^{1P}-H210$^{3.37}$, Tyr^{1P}-T213$^{3.40}$, Asp^{3P}-K182$^{2.67}$, Phe^{6P}-F126$^{1.36}$, Asn^{8P}-D274$^{45.52}$, Asn^{8P}-D276^{ECL2}, Arg^{11P}-D274$^{45.52}$ and Arg^{11P}-H194^{ECL1}), different from other class B GPCR–peptide pairs, may be responsible for GHRH specificity. **d**, The peptide recognition modes are described by fingerprint strings encoding different interaction types of the surrounding residues in each receptor. Color codes are listed on the top panel. **e** and **f**, Diversified forms of peptide recognition among class B GPCRs. Despite a conserved Asp/Glu near the N terminus, peptides form receptor- and peptide-specific polar networks between TM1, TM2 and ECL2 as well as among themselves by adjusting distinct orientations of side chains. Five peptide-bound receptor-G protein complexes were adopted in peptide-binding mode analysis: LA-PTH-bound PTH1R (PDB: 6NBF), GLP-1-bound GLP-1R (PDB: 5VAI), ExP5-bound GLP-1R (PDB: 6B3J), glucagon-bound GCGR (PDB: 6LMK) and PACAP38-bound PAC1R (PDB: 6P9Y).

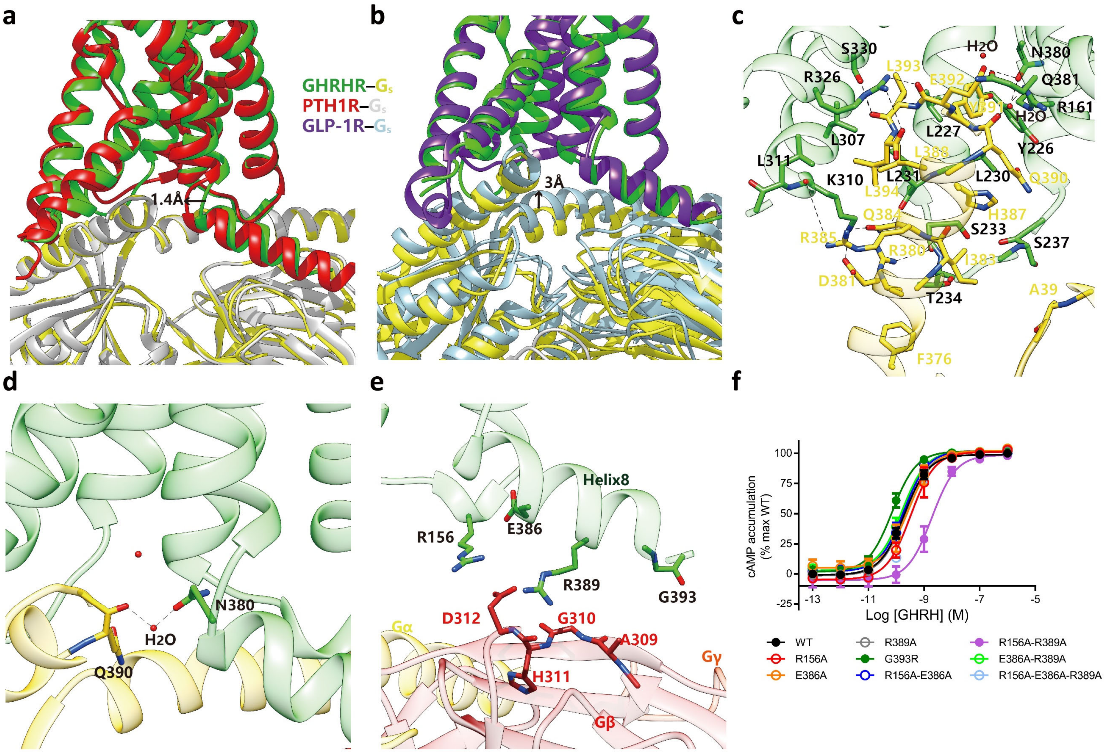

Supplementary Figure 9. Structure comparison of GHRHR–G_s interface with that of other class B GPCRs. a and **b,** comparison of interaction mode between different class B GHRHR–G_s complexes. GHRHR–G_s, lime green-yellow; PTH1R–G_s, grey-light green; GLP-1R–G_s, gold-cyan. **c,** GHRHR–G_s interface, including Gαs α5-GHRHR helix 8 interface. **d,** the hydrogen bond between GHRHR and Gαs mediated by water. GHRHR, lime green; Gα_s, yellow; and Gβ, red. **e,** the electrostatic interaction network in the interface of GHRHR and Gβ. **f,** Representative effects of GHRHR-G_s interface mutations on the G_s-mediated cAMP accumulation. Data are presented as mean values ± S.E.M. of at least three independent experiments (n = 3–9), conducted in quadruplicate. Source data are provided as a Source Data file.

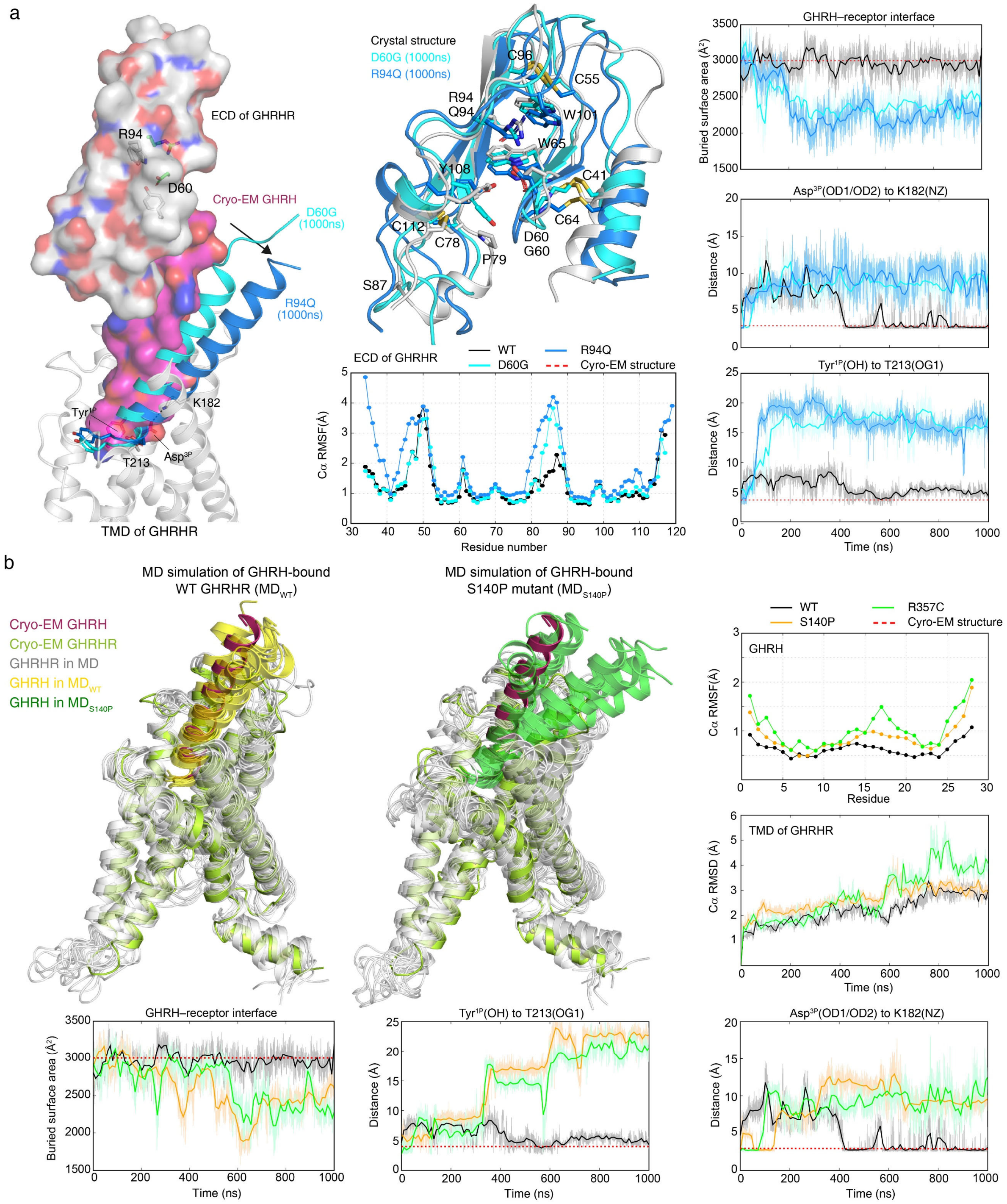

Supplementary Figure 10. Molecular dynamics (MD) simulation of GHRHR with disease-causing mutations. a, MD simulation of two disease-caused mutations on the GHRHR ECD, R94Q and D60G. The relative movement (root mean square deviation, RMSF) of the Cα positions of the GHRHR ECD, the buried surface area between GHRH and the receptor, and two distances between specified atoms from the residues on the N-terminal of GHRH and the receptor TMD region are shown. To evaluate the stability of ECD, all snapshots obtained from MD simulations were superimposed on the crystal structure of GHRHR ECD (PDB accession: 2XDG) and the last 500 ns trajectories were sent to RMSF

calculation. During the MD simulation of WT GHRHR, the N terminus of GHRH consistently interacted with the TMD core, in line with the cryo-EM structure, evidenced by the buried surface areas of GHRH-receptor interface and atomic distances of two pairs, Tyr^{1P}(OG1)–T213(OG1) and Asp^{3P}(OD1/OD2)-K182(NZ). The orientation of ECD relative to TMD was variable in the simulation, consistent with previous reports on PTH1R[12] and GLP-1R[8]. Compared to the WT, GHRHR mutants R94Q and D60G had a structurally less stable ECD because of the abolished salt bridge between R94 and D60. Consequently, the second and short α-helix (residues 79 to 87) was disordered and lost its compact with the C terminus of GHRH. The decreased interface area and elimination of essential interaction between the N terminus of GHRH and the receptor TMD core suggest that these two mutants may have both weakened binding affinity and reduced potency for GHRH. **b,** MD simulation of two disease-caused mutations on GHRHR TMD, S140P and R357C. MD snapshots were superimposed on the cryo-EM structure of GHRHR TMD using the Cα atoms of residues A120$^{1.30b}$–L154$^{1.64b}$ (TM1), C159$^{2.44b}$–L186$^{2.71b}$ (TM2), V200$^{3.27b}$–A232$^{3.59b}$ (TM3), R240$^{4.40b}$–F267$^{4.67b}$ (TM4), W282$^{5.36b}$–L311$^{5.65b}$ (TM5), Q323$^{6.34b}$–F347$^{6.58b}$ (TM6), and G355$^{7.36b}$–L379$^{7.60b}$ (TM7). Ten snapshots from 1 μs MD simulation trajectories were exacted to present the dynamic conformations of GHRHR (WT) and GHRHR mutant (S140P) and the receptor ECD was hidden. The relative movement of the Cα positions of GHRH, the root mean square deviation (RMSD) of the Cα positions of the GHRHR TMD, the buried surface area between GHRH and the receptor, and two distances between specified atoms from the residues on the N-terminal of GHRH and the receptor TMD region are shown. Different from the stable orientation of GHRH inserted into WT TMD core, mutants S120P and R357C first disrupted the salt bridge (Asp^{3P}-K182) and hydrogen bond (Tyr^{1P}–T213) with the N terminus of GHRH, then the C terminus of GHRH was released to twist, and finally, the overall buried surface between GHRH and the receptor decreased significantly, indicative of reduced ligand potency.

Supplementary Table 1. Effects of GHRH-mediated cAMP accumulation.

**Receptor**	**cAMP**		**Cell surface expression**	**Binding**
	**pEC_{50}**	**E_{max} (% WT)**	**(% WT)**	**pIC_{50}**
GHRHR(23-423) (WT)	9.20±0.10	100	100	8.56±0.17
GHRHR(23-405)	9.06±0.05	120.55±1.97*	86.97±6.28	ND
GHRHR(23-405)-15AA-LgBit-2MBP	8.54±0.13*	78.82±10.52*	53.92±6.37*	9.056±0.09

cAMP accumulation data were analyzed using a three-parameter logistic equation to determine pEC_{50} and E_{max} values. pEC_{50} is the negative logarithm of the molar concentration of agonist that induced half the maximal response. Cell surface expression was assessed by FACS to detect the N-terminal Flag epitope label [Anti-Flag antibody (Sigma-Aldrich, 1:300); Donkey anti-Mouse Alexa Fluor 488-conjugated secondary antibody, (ThermoFisher Scientific, 1:1000)] on the receptor and normalized to the wild-type (WT) GHRHR (shown as percentage). Binding data were analyzed using a three-parameter logistic equation to determine pIC_{50} values. All data shown are means ± S.E.M. of at least three independent experiments. One-way ANOVA and Dunnett's post-test were used to determine statistical difference. *P<0.01; ND, not determined.

Supplementary Table 2. Cryo-EM data collection, model refinement and validation statistics.

	GHRH–GHRHR–G_s
**Data collection and processing**	
Magnification	4,9310
Voltage (kV)	300
Electron exposure (e^-/Å^2)	62
Defocus range (μm)	-0.5 ~ -2.5
Pixel size (Å)	1.014
Symmetry imposed	C1
Initial particle projections (no.)	2,586,606
Final particle projections (no.)	307,018
Map resolution (Å)	2.6
FSC threshold	0.143
Map resolution range (Å)	2.1-4.0
**Refinement**	
Initial model used	6NBF
Model resolution (Å)	2.7
FSC threshold	0.143
Model resolution range (Å)	2.2-4.0
Map sharpening *B* factor (Å^2)	-99.27
Model composition	
Non-hydrogen atoms	8337
Protein residues	1046
Water	3
Lipids	3
B factors (Å^2)	
Protein	60.58
Water	67.38
Lipids	68.86
RMSD	
Bond lengths (Å)	0.004
Bond angles (°)	0.622
Validation	
MolProbity score	1.54
Clashscore	6.26
Rotamer outliers (%)	0.00
Ramachandran plot	
Favored (%)	96.78
Allowed (%)	3.22
Disallowed (%)	0

RMSD, root-mean-square deviation.

Supplementary Table 3. Interaction of GHRH N-terminal helix with TMD of GHRHR.

GHRH^{13}	GHRHR	Interaction
Tyr1	His210$^{3.37b}$	Side chain -side chain hydrogen bond
	Thr213$^{3.40b}$	Hydrophobic interaction
	Trp282$^{5.36b}$	
	Glu361$^{7.42b}$	Backbone-side chain hydrogen bond
Ala2	Arg357$^{7.38b}$	Hydrophobic interaction
	Leu358$^{7.39b}$	
	Glu361$^{7.42b}$	
	Leu362$^{7.43b}$	
Asp3	Val179$^{2.64b}$	Hydrophobic interaction
	Lys182$^{2.67b}$	Electrostatic interaction
Ile5	Asp350^{ECL3}	Hydrophobic interaction
	Leu354$^{7.35b}$	
	Arg357$^{7.38b}$	
Phe6	Phe126$^{1.36b}$	Hydrophobic interaction
	Val129$^{1.39b}$	
	Tyr133$^{1.43b}$	
	Leu362$^{7.43b}$	
Thr7	Lys182$^{2.67b}$	Side chain -side chain hydrogen bond
Asn8	Asp276^{ECL2}	Side chain -side chain hydrogen bond
	Asp274^{ECL2}	Backbone-side chain hydrogen bond
Ser9	Phe126$^{1.36b}$	Hydrophobic interaction
Tyr10	Phe126$^{1.36b}$	Hydrophobic interaction
	Phe187^{ECL1}	π-π stack
Arg11	Phe187^{ECL1}	Hydrophobic interaction
	His194^{ECL1}	Side chain -side chain hydrogen bond<br>Backbone-side chain hydrogen bond
	Ala271^{ECL2}	Backbone-side chain hydrogen bond
	Cys272 ^{ECL2}	Hydrophobic interaction
	Asp274 ^{ECL2}	Electrostatic interaction
Val13	Glu122$^{1.32b}$	Side chain -side chain hydrogen bond
Leu14	His194 ^{ECL1}	Hydrophobic interaction
Gly15	His194 ^{ECL1}	Hydrophobic interaction
	Cys195 ^{ECL1}	
Gln16	Leu119$^{1.29b}$	Hydrophobic interaction
Leu17	Leu119$^{1.29b}$	Hydrophobic interaction
	Glu123$^{1.33b}$	
Ser18	Asp193^{ECL1}	Backbone-side chain hydrogen bond

Residues within 4 Å are shown.

Supplementary Table 4. Effects of residue mutation in the ligand-binding pocket on GHRH-induced cAMP accumulation.

Mutants	cAMP		Cell surface expression (% WT)
	pEC_{50}±SEM	E_{max} (% WT)	
Wild-type	9.74±0.17	100	100
L119A	9.14±0.05	98.67±1.48	87.93±3.90
E122A	9.96±0.15	98.38±3.26	75.6±4.21
E123A	10.14±0.06	100.33±1.34	100.1±5.86
F126A	8.96±0.12*	98.92±0.84	91.04±6.38
V129A	6.78±0.08*	24.33±6.11*	1.75±0.50*
V179A	9.54±0.28	101.24±3.06	95.09±7.62
K182A	7.40±0.06*	100.02±1.40	43.75±6.15*
F187A	8.94±0.26*	101.68±1.52	120.7±3.89
T192A	9.64±0.03	103.11±1.18	104.2±6.58
D193A	9.94±0.20	101.42±1.02	99.3±4.76
H194A	9.60±0.04	98.01±1.25	93.96±5.54
C195A	7.67±0.06*	95.22±1.05	84.21±6.09
S209A	9.96±0.03	100.52±0.43	88.94±9.95
H210A	9.00±0.19	99.66±1.10	50.06±5.88*
T213A	9.35±0.06	91.48±4.55	27.22±5.06*
A271R	9.98±0.12	100.81±1.19	97.44±7.87
A271F	9.73±0.19	103.70±0.64	69.29±4.50*
D274A	9.33±0.04	99.78±2.32	76.04±7.79
D276A	9.48±0.06	102.04±0.51	95.71±6.47
W282A	7.88±0.24*	101.88±5.62	126.3±6.195
K286A	8.81±0.21*	98.61±1.91	98.63±19.65
I285A	7.18±0.34*	102.00±0.20	6.44±0.66*
I289A	8.01±0.17*	93.36±2.36	5.59±0.85*
N346A	8.85±0.27*	101.21±3.69	97.97±4.84
D350A	6.83±0.13*	47.38±8.48*	8.78±2.69*
L354A	9.74±0.10	98.79±1.84	105±7.02
R357A	6.87±0.09*	60.05±5.19*	100.9±8.42
L358A	8.98±0.13	100.3±1.21	121±8.64
E361A	8.98±0.23	18.75±4.38*	8.70±0.92*
L362A	9.95±0.15	103.97±0.47	125.10±33.92

cAMP accumulation data were analyzed using a three-parameter logistic equation to determine pEC_{50} and E_{max} values. E_{max} values for mutants are expressed as a percentage of the wild-type (WT). Cell surface expression was assessed by FACS to detect the N-terminal Flag epitope label on the receptor and normalized to the WT GHRHR (shown as percentage). All data are means ± S.E.M. of at least three independent experiments. One-way ANOVA was used to determine statistical significance (*P< 0.01). NS, not saturable.

Supplementary Table 5. Summary of disease-causing missense mutations in GHRHR.

Variant ID or PMID	Mutation	Genotype	Position	Disease	Validated functional data
rs139599160	V10G	Heterozygous	N-terminus	IGHD[14]	Lower GHRHR gene expression at cellular surface[14]
16959974	A45T	Homozygous	N-terminus	Colorectal cancer[15]	NR
8391647	D60G (mouse)	Homozygous	N-terminus	*Little* mouse[16, 17]	Functionally defective and unable to transduce GRF-dependent increases in intracellular cAMP levels[16]
rs1319200922	C64G	Homozygous	N-terminus	IGHD[18]	NR
rs775025721	S70A	Unknown[a]	N-terminus	IGHD[b]	NR
rs121918117	E72K	Homozygous	N-terminus	IGHD	NR
rs776859854	P77L	Unknown[a]	N-terminus	Not provided[c]	NR
rs765640577	P79L	Homozygous	N-terminus	IGHD[19]	Reduction in activity and the altered affinity[19]
rs200848306	R94Q	Homozygous	N-terminus	IGHD[20]	Complete loss of function[20]
rs376258046	R94L	Homozygous	N-terminus	IGHD[21]	NR
rs376258046	R94W	Heterozygous	N-terminus	IGHD[22]	NR
30266296	C112Y	Heterozygous	N-terminus	IGHD[23]	NR
rs4988498	E121D	Homozygous	1.31	IGHD[14, 24]	Close to WT
23602557	G136V	Heterozygous	1.46	IGHD[25]	Elicited no luciferase activity increment in response to GHRH stimulation, with normal membrane expression[25]
rs1311381263	H137L	Heterozygous	1.47	IGHD[26]	Failed to increase cAMP after treatment with GHRH[26, 27]
rs606231412	S140P	Homozygous[b]	1.50	IGHD[d]	NR
rs121918118	L144H	Homozygous	1.54	IGHD[20, 28]	Failed to show a cAMP response after treatment of the cells with GHRH[20, 27, 28, 29]
rs779187338	A153D	Homozygous	1.63	IGHD[20]	Complete loss of function[20]
rs758798716	R161W	Homozygous	2.46	IGHD[20, 21]	Complete loss of function[20]
rs746565662	N162I	Unknown[a]	2.47	IGHD[b]	NR
rs886043578	N162D	Heterozygous[b]	2.47	Not provided[c]	NR
rs1045584744	V164A	Homozygous	2.49	IGHD[20]	Complete loss of function[20]
rs606231413	H165Q	Heterozygous[b]	2.50	IGHD[d]	NR
rs570281194	F169L	Heterozygous	2.54	IGHD[20]	Partial but significant loss of function[20]
rs10227922	T171S	Heterozygous	2.56	IGHD[b,d]	NR
rs774281185	A176V	Homozygous	2.61	IGHD[20, 30, 31]	Significantly reduced cAMP response[20, 27, 30]
rs765740795	A184P	Heterozygous	2.69	Not provided[c]	NR
rs535947130	M214V	Unknown[a]	3.41	IGHD[b]	NR
rs121918120	A222E	Homozygous	3.49	IGHD[21, 28]	Failed to show a cAMP response after treatment of the cells with GHRH[27, 28]

rs28371560	V225I	Homozygous	3.52	GH-producing pituitary tumors[24]	Reduced GHRH binding and similar cAMP elevation as WT[24]
rs121918119	F242C	Heterozygous	4.42	IGHD[28]	Failed to show a cAMP response after treatment of the cells with GHRH[27, 28]
rs1004753042	P253L	Heterozygous	4.53	IGHD[23]	NR
25541890	T257A	Homozygous	4.57	IGHD[32]	NR
31231873	T259K	Heterozygous	4.59	IGHD[20]	Complete loss of function[20]
25541890	K264E	Homozygous	4.64	IGHD[32]	NR
rs547906129	A271V	Unknown[a]	ECL2	IGHD[b]	NR
19567534	W273S	Homozygous	ECL2	IGHD[21]	NR
rs1361718232	W283R	Homozygous	5.37	IGHD[20]	Complete loss of function[20]
rs527387367	S292W	Unknown[a]	5.46	IGHD[b]	NR
rs748546851	G294R	Heterozygous	5.48	GH-producing pituitary tumors[24]	No cAMP stimulation by GHRH[24]
rs200472991	R305H	Unknown[a]	5.59	IGHD[b]	NR
25541890	S317T	Heterozygous	ICL3	IGHD[32]	NR
rs121918121	K329E	Heterozygous	6.40	IGHD[33]	Failed to show a cAMP response after treatment with GHRH[27, 33]
rs752122561	S330L	Homozygous	6.41	IGHD[32]	NR
rs149182247	P336L	Unknown[a]	6.47	Not provided[c]	NR
rs794727020	D350G	Heterozygous	ECL3	Not provided[c]	NR
rs376948691	R357C	Homozygous	7.38	IGHD[29]	Complete inactivity *in vitro*[29]
25541890	G369V	Homozygous	7.50	IGHD[32]	NR
31231873	I387T	Homozygous	8.54	IGHD[20]	Complete loss of function[20]
rs2228078	M422T	Heterozygous	C-term	IGHD[b]	NR

[a]The zygosity information of the disease-causing mutation in ClinVar is not provided.

[b]The mutation submitted by Illumina Clinical Services Laboratory.

[c]The disease information associated with the mutation in ClinVar is not provided.

[d]The mutation submitted by Endocrinology Clinic, Seth G.S. Medical College.

To date, 49 human GHRHR disease-causing natural missense mutations were reported (Fig. 4A), and 41 of them are either caused or associated with IGHD, a disease that affects the production, release and functional activity of GH leading to short stature. Based on the occurring positions, these mutations were mapped into the GHRHR structure with four classes: ECD region (11 mutations), ligand-binding pocket (11 mutations), G protein coupling region (13 mutations), and the central region connecting ligand-binding and G protein-coupling (the connector, 14 mutations). To collect disease-causing mutations for GHRHR, we performed database integration (Uniport, OMIM, Ensembl, ClinVar and HGMD) and literature investigation. In addition to the missense mutants described in the text, several assumed disease-causing mutations with undefined clinical significance, such as A271V and S292W, were also studied which neither affected GHRH-induced cAMP accumulation nor influenced β-arrestin2 recruitment, implying that they might be part of GHRHR polymorphism. WT, wild-type; IGHD, isolated growth hormone deficiency; NR, not reported. Orange shadow rows indicate missense mutants that were functionally studied and four of them marked in red were evaluated with MD simulations.

Supplementary Table 6. List of primers sequences for site-direct mutagenesis studies, related to Figs. 2, 4, 5, Supplementary Figs. 6, 8 and Supplementary Tables 4, 5.

**Oligonucleotide name**	**Oligonucleotide sequence (5'-3')**	**Cloning method**	**Product**
GHRHR(23-405)-forward	TCTTCTGCCTGGTATTCGCCCACATGCACCCAGAATGTGACT	Homologous recombination	pFastBac-GHRHR(23-405)
GHRHR(23-405)-reverse	TACAGATTCTCTGAACCTCCACGGGTCCTCCAGGCTGG		
Linear-pFastBac-forward	GGAGGTTCAGAGAATCTGTACTTCCA		
Linear-pFastBac-reverse	GGCGAATACCAGGCAGAAGA		
GHRHR(23-405)-LgBiT-forward	TCTTCTGCCTGGTATTCGCCCACATGCACCCAGAATGTGACT		pFastBac-GHRHR(23-405)-15AA-LgBiT
GHRHR(23-405)-LgBiT-reverse	CCGCCACCACCGCTCGAGCCACGGGTCCTCCAGGCTGG		
Linear-pFastBac-LgBiT-forward	GGCTCGAGCGGTGGTGGC		
Linear-pFastBac-LgBiT-reverse	GGCGAATACCAGGCAGAAGA		
pBiT-GHRHR(23-405)-forward	CCACAGGTGTCCACTCCGAGCACATGCACCCAGAATGTGACT	Homologous recombination	pBiT-GHRHR(23-405)-LgBiT & pBiT-GHRHR(23-423)-LgBiT
pBiT-GHRHR(23-405)-reverse	AAATCTTCGAGTGTGAAGACACGGGTCCTCCAGGCTGG		
pBiT-GHRHR(23-423)-forward	CCACAGGTGTCCACTCCGAGCACATGCACCCAGAATGTGACT		
pBiT-GHRHR(23-423)-reverse	AAATCTTCGAGTGTGAAGACGCACATAGATGTCAGCACCTTTG		
Linear-pBiT-forward	GTCTTCACACTCGAAGATTTCGTTG		
Linear-pBiT-reverse	CTCGGAGTGGACACCTGTGG		
pBiT-GHRHR(23-405)-LgBiT-forward	CCACAGGTGTCCACTCCGAGCACATGCACCCAGAATGTGACT		pBiT-GHRHR(23-405)-15AA-LgBiT
pBiT-GHRHR(23-405)-LgBiT-reverse	CCGCCACCACCGCTCGAGCCACGGGTCCTCCAGGCTGG		
Linear-pBiT-LgBiT-forward	GGCTCGAGCGGTGGTGGC		
Linear-pBiT-LgBiT-reverse	CTCGGAGTGGACACCTGTGG		
pcDNA3.1-	CCGGCAGCGCCGGCAGCGCCCACATGCAC	Homologous	pcDNA3.1-

GHRHR(23-405)-forward	CCAGAATGTGACT	recombination	GHRHR(23-405) & pcDNA3.1-GHRHR(23-423) & pcDNA3.1-GHRHR(23-405)-15AA-LgBiT-2MBP
pcDNA3.1-GHRHR(23-405)-reverse	TGCTGGATATCTGCAGAATTCTAACGGGTCCTCCAGGCTG		
pcDNA3.1-GHRHR(23-423)-forward	CCGGCAGCGCCGGCAGCGCCCACATGCACCCAGAATGTGACT		
pcDNA3.1-GHRHR(23-423)-reverse	TGCTGGATATCTGCAGAATTCTAGCACATAGATGTCAGCACCTTT		
pcDNA3.1-GHRHR(23-405)-LgBiT-forward	CCGGCAGCGCCGGCAGCGCCCACATGCACCCAGAATGTGACT		
pcDNA3.1-GHRHR(23-405)-LgBiT-reverse	GGATATCTGCAGAATTCTTACTTGGTGATACGAGTCTGCGC		
Linear-pcDNA3.1-forward	TAAGAATTCTGCAGATATCCAGCAC		
Linear-pcDNA3.1-reverse	GGCGCTGCCGGCGCTGCC		
pcDNA3.1-GHRHR(23-423)-Rluc8-forward	CCGGCAGCGCCGGCAGCGCCCACATGCACCCAGAATGTGACT	Homologous recombination	pcDNA3.1-GHRHR(23-423)-Rluc8
pcDNA3.1-GHRHR(23-423)-Rluc8-reverse	TTGTACAAGAAAGCTGGGTCGCACATAGATGTCAGCACCTTTG		
Linear-pcDNA3.1-Rluc8-forward	GACCCAGCTTTCTTGTACAAAGTG		
Linear-pcDNA3.1-Rluc8-reverse	GGCGCTGCCGGCGCTGCC		
L119A-forward	TGGCGGCTGAGGAGGAATCTTACTTCTCCACA	Site-directed mutagenesis	pcDNA3.1-GHRHR(23-423)-L119A
L119A-reverse	TTCCTCCTCAGCCGCCAGCTCCAGAGGCACAGGG		
E122A-forward	GCTGAGGCGGAATCTTACTTCTCCACAGTGAAGATTAT		pcDNA3.1-GHRHR(23-423)-E122A
E122A-reverse	TAAGATTCCGCCTCAGCCAGCAGCTCCAGAGG		
E123A-forward	TGAGGAGGCATCTTACTTCTCCACAGTGAAGATTATCTAC		pcDNA3.1-GHRHR(23-423)-E123A
E123A-reverse	AGTAAGATGCCTCCTCAGCCAGCAGCTCCAGA		
F126A-forward	ATCTTACGCCTCCACAGTGAAGATTATCTA		pcDNA3.1-

	CACCG		GHRHR(23-423)-F126A
F126A-reverse	CTGTGGAGGCGTAAGATTCCTCCTCAGCCAGCA		
V129A-forward	CTCCACAGCGAAGATTATCTACACCGTGGGCC		pcDNA3.1-GHRHR(23-423)-V129A
V129A-reverse	TAATCTTCGCTGTGGAGAAGTAAGATTCCTCCTCA		
V179A-forward	CTGCGTTCCTGAAGGATGCTGCCCTTTTCCAC		pcDNA3.1-GHRHR(23-423)-V179A
V179A-reverse	ATCCTTCAGGAACGCAGCTCCCGCCTTGAGGATAA		
K182A-forward	TGTTCCTGGCGGATGCTGCCCTTTTCCACAGC		pcDNA3.1-GHRHR(23-423)-K182A
K182A-reverse	AGCATCCGCCAGGAACACAGCTCCCGCCTTGA		
F187A-forward	TGCTGCCCTTGCCCACAGCGACGACACTGACCA		pcDNA3.1-GHRHR(23-423)-F187A
F187A-reverse	TGTGGGCAAGGGCAGCATCCTTCAGGAACACA		
T192A-forward	GCTGACCACTGCAGCTTCTCCACTGTTCTATG		pcDNA3.1-GHRHR(23-423)-T192A
T192A-reverse	AAGCTGCAGTGGTCAGCGTCGTCGCTGTGGAAAAGGG		
D193A-forward	ACTGCCCACTGCAGCTTCTCCACTGTTCTATG		pcDNA3.1-GHRHR(23-423)-D193A
D193A-reverse	AAGCTGCAGTGGGCAGTGTCGTCGCTGTGGAAAAG		
H194A-forward	ACTGACGCCTGCAGCTTCTCCACTGTTCTATGC		pcDNA3.1-GHRHR(23-423)-H194A
H194A-reverse	AAGCTGCAGGCGTCAGTGTCGTCGCTGTGGAA		
C195A-forward	TGACCACGCCAGCTTCTCCACTGTTCTATGCAAG		pcDNA3.1-GHRHR(23-423)-C195A
C195A-reverse	AGAAGCTGGCGTGGTCAGTGTCGTCGCTGTGG		
S209A-forward	GCCCATTTCGCCACCATGACCAACTTCAGCTG		pcDNA3.1-GHRHR(23-423)-S209A
S209A-reverse	ATGGTGGCGAAATGGGCGGCGGCCACAGAGACCTT		
H210A-forward	TCCGCTTTCGCCACCATGACCAACTTCAGCTG		pcDNA3.1-GHRHR(23-423)-H210A
H210A-reverse	ATGGTGGCGAAAGCGGAGGCGGCCACAGAGAC		

T213A-forward	CGCCATGACCAACTTCAGCTGGCTGTTGGCAG		pcDNA3.1-GHRHR(23-423)-T213A
T213A-reverse	TGAAGTTGGTCATGGCGGCGAAATGGGAGGCGGC		
A271R-forward	TTCGAGGACATCCGGTGCTGGGACCTGGACGACA		pcDNA3.1-GHRHR(23-423)-A271A
A271R-reverse	CACCGGATGTCCTCGAAGGCCAGTTTGCAGCT		
A271F-forward	GGACATCTTTTGCTGGGACCTGGACGACACCT		pcDNA3.1-GHRHR(23-423)-A271F
A271F-reverse	CCCAGCAAAAGATGTCCTCGAAGGCCAGTTTG		
D274A-forward	TGCTGGGCCCTGGACGACACCTCCCCCTACTG		pcDNA3.1-GHRHR(23-423)-D274A
D274A-reverse	TCGTCCAGGGCCCAGCACGCGATGTCCTCGAA		
D276A-forward	GGACCTGGCCGACACCTCCCCCTACTGGTGGA		pcDNA3.1-GHRHR(23-423)-D276A
D276A-reverse	AGGTGTCGGCCAGGTCCCAGCACGCGATGTCC		
W282A-forward	CTACGCGTGGATCATCAAAGGGCCCATTGTCC		pcDNA3.1-GHRHR(23-423)-W282A
W282A-reverse	TGATGATCCACGCGTAGGGGGAGGTGTCGTCCA		
K286A-forward	ACTGGTGGATCGCCAAAGGGCCCATTGTCCTCTC		pcDNA3.1-GHRHR(23-423)-K286A
K286A-reverse	TTTGGCGATCCACCAGTAGGGGGAGGTGTCGT		
I285A-forward	ACTGGTGGATCATCGCAGGGCCCATTGTCCTCTCG		pcDNA3.1-GHRHR(23-423)-I285A
I285A-reverse	TGCGATGATCCACCAGTAGGGGGAGGTGTCGT		
I289A-forward	ATCAAAGGGCCCGCTGTCCTCTCGGTCGGGGTG		pcDNA3.1-GHRHR(23-423)-I289A
I289A-reverse	ACAGCGGGCCCTTTGATGATCCACCAGTAGGG		
N346A-forward	TCATCTTCGCCTTCCTGCCAGACAATGCTGGC		pcDNA3.1-GHRHR(23-423)-N346A
N346A-reverse	CAGGAAGGCGAAGATGATGTAGTGAATTCCAAAGAG		
D350A-forward	ACTTCCTGCCAGCCAATGCTGGCCTGGGCATC		pcDNA3.1-GHRHR(23-423)-D350A
D350A-reverse	ATTGGCTGGCAGGAAGTTGAAGATGATGTAGT		

L354A-forward	AATGCTGGCGCGGGCATCCGCCTCCCCCT GGA		pcDNA3.1-GHRHR(23-423)-L354A
L354A-reverse	ATGCCCGCGCCAGCATTGTCTGGCAGGAA GTT		
R357A-forward	ATCGCCCTCCCCCTGGAGCTGGGACTGGG TTC		pcDNA3.1-GHRHR(23-423)-R357A
R357A-reverse	TCCAGGGGGAGGGCGATGCCCAGGCCAGC ATT		
L358A-forward	ATCCGCGCCCCCCTGGAGCTGGGACTGGG TTC		pcDNA3.1-GHRHR(23-423)-L358A
L358A-reverse	TCCAGGGGGGCGCGGATGCCCAGGCCAGC ATT		
E361A-forward	TGGCGCTGGGACTGGGTTCCTTCCAGGGC TTC		pcDNA3.1-GHRHR(23-423)-E361A
E361A-reverse	ACCCAGTCCCAGCGCCAGGGGGAGGCGGA TGCC		
L362A-forward	GCGGGACTGGGTTCCTTCCAGGGCTTCAT TGT		pcDNA3.1-GHRHR(23-423)-L362A
L362A-reverse	AAGGAACCCAGTCCCGCCTCCAGGGGGAG GCGGAT		
R156A-forward	TCTCAGGGCGCTCCACTGCCCCCGGAACT ACG		pcDNA3.1-GHRHR(23-423)-R156A
R156A-reverse	AGTGGAGCGCCCTGAGAGCAACCAGGATG GTG		
E386A-forward	TGAGGACTGCGATCTCACGGAAGTGGCAT GGC		pcDNA3.1-GHRHR(23-423)-E386A
E386A-reverse	TGAGATCGCAGTCCTCACCTCTTGGTTGA GGA		
R389A-forward	AGATCTCAGCGAAGTGGCATGGCCATGAC CCT		pcDNA3.1-GHRHR(23-423)-R389A
R389A-reverse	CCACTTCGCTGAGATCTCAGTCCTCACCTC TTG		
G393R-forward	AAGTGGCATCGCCATGACCCTGAGCTTCT GCC		pcDNA3.1-GHRHR(23-423)-G393R
G393R-reverse	TCATGGCGATGCCACTTCCGTGAGATCTC AGT		
D60G-forward	ACCTGGGGTGGGCTGCTGTGCTGGCCAAC GGC		pcDNA3.1-GHRHR(23-423)-D60G
D60G-reverse	AGCAGCCCACCCCAGGTCGCAGGGCAGCC CAG		
E72K-forward	CTCTGGCAAGTGGGTCACCCTCCCCTGCC CGG		pcDNA3.1-GHRHR(23-423)-E72K
E72K-reverse	TGACCCACTTGCCAGAGCCTGCCGTTGGC CAG		

P77L-forward	CTCTGCCCGGATTTCTTCTCTCACTTCAGCTC		pcDNA3.1-GHRHR(23-423)-P77L & pcDNA3.1-GHRHR(23-423)-Rluc8-P77L
P77L-reverse	AAGAAATCCGGGCAGAGGAGGGTGACCCACTCGCC		
R94Q-forward	GCTGTGAAACAGGATTGTACTATCACTGGCTGGTCTG		pcDNA3.1-GHRHR(23-423)-R94Q & pcDNA3.1-GHRHR(23-423)-Rluc8-R94Q
R94Q-reverse	CAATCCTGTTTCACAGCCCCTGACTCTGAGCT		
S140P-forward	AGCATCCCTATTGTAGCCCTCTTCGTGGCCAT		pcDNA3.1-GHRHR(23-423)-S140P & pcDNA3.1-GHRHR(23-423)-Rluc8-S140P
S140P-reverse	GCTACAATAGGGATGCTATGGCCCACGGTGTA		
N162I-forward	ATCTACGTCCACACCCAGCTGTTCACCACTTT		pcDNA3.1-GHRHR(23-423)-N162I & pcDNA3.1-GHRHR(23-423)-Rluc8-N162I
N162I-reverse	TGGGTGTGGACGTAGATCCGGGGGCAGTGGAGCCT		
N162D-forward	GACTACGTCCACACCCAGCTGTTCACCACTTT		pcDNA3.1-GHRHR(23-423)-N162D & pcDNA3.1-GHRHR(23-423)-Rluc8-N162D
N162D-reverse	TGGGTGTGGACGTAGTCCCGGGGGCAGTGGAGCCT		
H165Q-forward	ACTACGTCCAAACCCAGCTGTTCACCACTTTTATC		pcDNA3.1-GHRHR(23-423)-H165Q & pcDNA3.1-GHRHR(23-423)-Rluc8-H165Q
H165Q-reverse	CTGGGTTTGGACGTAGTTCCGGGGGCAGTGGA		
T171S-forward	CAGCTGTTCACCTCTTTTATCCTCAAGGCGGGAGC		pcDNA3.1-GHRHR(23-423)-T171S & pcDNA3.1-GHRHR(23-423)-Rluc8-T171S
T171S-reverse	AAAGAGGTGAACAGCTGGGTGTGGACGTAGTT		
A176V-forward	TATCCTCAAGGTGGGAGCTGTGTTCCTGAAGGATG		pcDNA3.1-GHRHR(23-423)-

A176V-reverse	CTCCCACCTTGAGGATAAAAGTGGTGAACAGC		A176V & pcDNA3.1-GHRHR(23-423)-Rluc8-A176V
M214V-forward	CACCGTGACCAACTTCAGCTGGCTGTTGGCAG		pcDNA3.1-GHRHR(23-423)-M214V & pcDNA3.1-GHRHR(23-423)-Rluc8-M214V
M214V-reverse	TGAAGTTGGTCACGGTGGCGAAATGGGAGGCG		
A271V-forward	TTCGAGGACATCGTGTGCTGGGACCTGGACGACA		pcDNA3.1-GHRHR(23-423)-A271V & pcDNA3.1-GHRHR(23-423)-Rluc8-A271V
A271V-reverse	CACACGATGTCCTCGAAGGCCAGTTTGCAGCT		
S292W-forward	ATTGTCCTCTGGGTCGGGGTGAACTTTGGGCT		pcDNA3.1-GHRHR(23-423)-S292W & pcDNA3.1-GHRHR(23-423)-Rluc8-S292W
S292W-reverse	CCGACCCAGAGGACAATGGGCCCTTTGATGAT		
P336L-forward	CCTGATCCTACTCTTTGGAATTCACTACATCATCTTC		pcDNA3.1-GHRHR(23-423)-P336L & pcDNA3.1-GHRHR(23-423)-Rluc8-P336L
P336L-reverse	CAAAGAGTAGGATCAGGAAAAGTGTCGACTTGG		
R357C-forward	ATCTGCCTCCCCCTGGAGCTGGGACTGGGTTC		pcDNA3.1-GHRHR(23-423)-R357C & pcDNA3.1-GHRHR(23-423)-Rluc8-R357C
R357C-reverse	TCCAGGGGGAGGCAGATGCCCAGGCCAGCATT		
119-423-forward	CTGGCTGAGGAGGAATCTTACTTCTCCACAGTG		pcDNA3.1-GHRHR(119-423)
119-423-reverse	AGATTCCTCCTCAGCCAGGGCGCTGCCGGCGCTGCC		

References

1. Hall MP, *et al.* Engineered luciferase reporter from a deep sea shrimp utilizing a novel imidazopyrazinone substrate. *ACS Chem Biol* **7**, 1848-1857 (2012).
2. Dixon AS, *et al.* NanoLuc complementation reporter optimized for accurate measurement of protein interactions in cells. *ACS Chem Biol* **11**, 400-408 (2016).
3. Inoue A, *et al.* Illuminating G-protein-coupling selectivity of GPCRs. *Cell* **177**, 1933-1947.e1925 (2019).
4. Duan J, *et al.* Cryo-EM structure of an activated VIP1 receptor-G protein complex revealed by a NanoBiT tethering strategy. *Nat Commun* (2020).
5. Zhang Y, *et al.* Cryo-EM structure of the activated GLP-1 receptor in complex with a G protein. *Nature* **546**, 248-253 (2017).
6. Liang YL, *et al.* Phase-plate cryo-EM structure of a biased agonist-bound human GLP-1 receptor-Gs complex. *Nature* **555**, 121-125 (2018).
7. Jazayeri A, *et al.* Crystal structure of the GLP-1 receptor bound to a peptide agonist. *Nature* **546**, 254-258 (2017).
8. Wu F, *et al.* Full-length human GLP-1 receptor structure without orthosteric ligands. *Nat Commun* **11**, 1272 (2020).
9. Zhou Q, *et al.* Common activation mechanism of class A GPCRs. *Elife* **8**, 31 (2019).
10. Wootten D, Simms J, Miller LJ, Christopoulos A, Sexton PM. Polar transmembrane interactions drive formation of ligand-specific and signal pathway-biased family B G protein-coupled receptor conformations. *Proc Natl Acad Sci U S A* **110**, 5211-5216 (2013).
11. Isberg V, *et al.* Generic GPCR residue numbers - aligning topology maps while minding the gaps. *Trends Pharmacol Sci* **36**, 22-31 (2015).
12. Zhao LH, *et al.* Structure and dynamics of the active human parathyroid hormone receptor-1. *Science* **364**, 148-153 (2019).
13. Pal K, Melcher K, Xu HE. Structure and mechanism for recognition of peptide hormones by Class B G-protein-coupled receptors. *Acta Pharmacol Sin* **33**, 300-311 (2012).
14. Godi M, *et al.* A recurrent signal peptide mutation in the growth hormone releasing hormone receptor with defective translocation to the cell surface and isolated growth hormone deficiency. *J Clin Endocrinol Metab* **94**, 3939-3947 (2009).
15. Sjoblom T, *et al.* The consensus coding sequences of human breast and colorectal cancers. *Science* **314**, 268-274 (2006).
16. Lin SC, Lin CR, Gukovsky I, Lusis AJ, Sawchenko PE, Rosenfeld MG. Molecular basis of the little mouse phenotype and implications for cell type-specific growth. *Nature* **364**, 208-213 (1993).
17. Godfrey P, Rahal JO, Beamer WG, Copeland NG, Jenkins NA, Mayo KE. GHRH receptor of little mice contains a missense mutation in the extracellular domain that disrupts receptor function. *Nature Genet* **4**, 227-232 (1993).
18. Demirbilek H, *et al.* Familial isolated growth hormone deficiency due to a novel homozygous missense mutation in the growth hormone releasing hormone receptor gene: clinical presentation with hypoglycemia. *J Clin Endocrinol Metab* **99**, E2730-2734 (2014).
19. Gregory LC, *et al.* Partial loss of function of the GHRH receptor leads to mild growth hormone deficiency. *J Clin Endocrinol Metab* **101**, 3608-3615 (2016).
20. Cohen E, *et al.* Contribution of functionally assessed GHRHR mutations to idiopathic isolated

growth hormone deficiency in patients without GH1 mutations. *Hum Mutat* **40**, 2033-2043 (2019).

21. Alatzoglou KS, *et al.* Expanding the spectrum of mutations in GH1 and GHRHR: genetic screening in a large cohort of patients with congenital isolated growth hormone deficiency. *J Clin Endocrinol Metab* **94**, 3191-3199 (2009).
22. Tommiska J, Jorgensen N, Christiansen P, Juul A, Raivio T. A homozygous R262Q mutation in the gonadotropin-releasing hormone receptor presenting as reversal of hypogonadotropic hypogonadism and late-onset hypogonadism. *Clin Endocrinol (Oxf)* **78**, 316-317 (2013).
23. Blum WF, *et al.* Screening a large pediatric cohort with GH deficiency for mutations in genes regulating pituitary development and GH secretion: Frequencies, phenotypes and growth outcomes. *EBioMedicine* **36**, 390-400 (2018).
24. Lee EJ, *et al.* Absence of constitutively activating mutations in the GHRH receptor in GH-producing pituitary tumors. *J Clin Endocrinol Metab* **86**, 3989-3995 (2001).
25. Soneda A, *et al.* Novel compound heterozygous mutations of the growth hormone-releasing hormone receptor gene in a case of isolated growth hormone deficiency. *Growth Horm IGF Res* **23**, 89-97 (2013).
26. Salvatori R, Fan X, Phillips JA, 3rd, Prince M, Levine MA. Isolated growth hormone (GH) deficiency due to compound heterozygosity for two new mutations in the GH-releasing hormone receptor gene. *Clin Endocrinol (Oxf)* **54**, 681-687 (2001).
27. Alba M, Salvatori R. Naturally-occurring missense mutations in the human growth hormone-releasing hormone receptor alter ligand binding. *J Endocrinol* **186**, 515-521 (2005).
28. Salvatori R, *et al.* Three new mutations in the gene for the growth hormone (gh)-releasing hormone receptor in familial isolated gh deficiency type ib. *J Clin Endocrinol Metab* **86**, 273-279 (2001).
29. Haskin O, *et al.* A new mutation in the growth hormone-releasing hormone receptor gene in two Israeli Arab families. *J Endocrinol Invest* **29**, 122-130 (2006).
30. Carakushansky M, *et al.* A new missense mutation in the growth hormone-releasing hormone receptor gene in familial isolated GH deficiency. *Eur J Endocrinol* **148**, 25-30 (2003).
31. Marui S, *et al.* GH-releasing hormone receptor gene: a novel splice-disrupting mutation and study of founder effects. *Horm Res Paediatr* **78**, 165-172 (2012).
32. Arman A, Dundar BN, Cetinkaya E, Erzaim N, Buyukgebiz A. Novel growth hormone-releasing hormone receptor gene mutations in Turkish children with isolated growth hormone deficiency. *J Clin Res Pediatr Endocrinol* **6**, 202-208 (2014).
33. Salvatori R, Fan XG, Mullis PE, Haile A, Levine MA. Decreased expression of the GHRH receptor gene due to a mutation in a Pit-1 binding site. *Mol Endocrinol* **16**, 450-458 (2002).
